# Colorectal Cancer: Epidemiology, Risk Factors, Signaling Pathways, Clinical Features, Screening, Diagnosis, and Management

**DOI:** 10.1002/mco2.71028

**Published:** 2026-10-02

**Authors:** Kairui Wan, Ke Tang, Linglong Wang, Qin Tian, Mingwei Zheng, Cheng Qian, Limei Liu

**Affiliations:** ^1^ Chongqing Key Laboratory of Translational Research for Cancer Metastasis and Individualized Treatment, Chongqing University Cancer Hospital, School of Medicine Chongqing University Chongqing China

**Keywords:** colorectal cancer, early‐onset colorectal cancer, precision oncology, screening, signaling pathways, tumor microenvironment

## Abstract

Colorectal cancer (CRC) remains a leading cause of cancer incidence and mortality worldwide and is increasingly characterized by two converging realities: a substantial global burden and a rising incidence of early‐onset CRC. This review synthesizes current advances in CRC epidemiology, risk factors, signaling biology, clinical presentation, screening, diagnosis, and management across the care continuum. We frame CRC as a systems‐level disease in which genomic instability, epigenetic dysregulation, metabolic remodeling, tumor microenvironmental evolution, and gut microbiota interactions converge on interconnected pathways, including Wnt/β‐catenin, MAPK, PI3K/AKT/mTOR, TGF‐β, and immune‐regulatory networks. We distinguish established standards of care from recent practice‐changing developments and investigational strategies. Within this framework, we discuss blood‐based screening, AI‐assisted endoscopy, ctDNA‐informed residual disease assessment, biomarker‐defined molecular workup, immunotherapy strategies for dMMR disease, and targeted approaches for KRAS G12C‐mutated, BRAF V600E‐mutated, and HER2‐positive metastatic CRC. Despite substantial progress, major challenges remain, including limited immunotherapy benefit in microsatellite‐stable CRC, unequal access to high‐quality screening and molecular testing, and uncertainty regarding implementation of newer biomarkers across diverse populations. An integrated framework linking population risk, tumor ecology, evidence‐based biomarker use, and equitable precision care is essential for the next phase of CRC prevention and management.

## Graphical abstract

Colorectal cancer is a heterogeneous, adaptive disease shaped by population‐level risk, genomic and epigenetic alterations, tumor ecosystem evolution, and treatment pressure. This review integrates epidemiology, risk factors, signaling networks, clinical presentation, screening, molecular diagnosis, and stage‐ and biomarker‐guided management while distinguishing current standards, practice‐changing advances, and unresolved translational challenges.

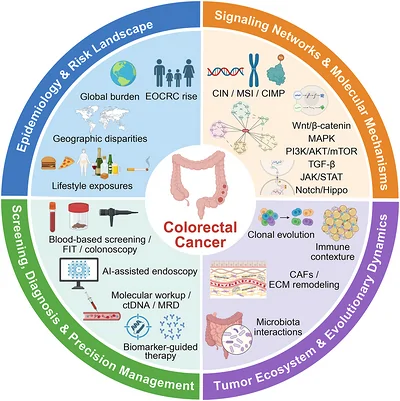

## 1 Introduction

Colorectal cancer (CRC) remains a major contributor to global cancer incidence and mortality and has long served as a model disease for multistep carcinogenesis [1, 2, 3]. Classical descriptions of the adenoma–carcinoma sequence established the idea that colorectal tumorigenesis unfolds through cumulative molecular alterations over time [4, 5]. Although that framework remains foundational, contemporary CRC research has shown that it is insufficient to explain the biological diversity, clinical heterogeneity, and treatment resistance observed in modern practice [6, 7, 8].

High‐throughput sequencing, single‐cell analysis, spatial profiling, and microbiome research have recast CRC as a heterogeneous, adaptive, and multiscale disease [7, 9, 10]. Population‐based screening has reduced incidence and mortality in many older adults, yet the absolute global burden remains high because of demographic aging, unequal access to care, and persistent exposure to modifiable risks [1, 2, 11]. At the same time, early‐onset CRC (EOCRC) has emerged as a major challenge, prompting investigation into how life‐course environmental, metabolic, inflammatory, and microbial exposures interact with inherited susceptibility and birth‐cohort effects. The relative contribution and causal importance of these factors remain incompletely defined [10, 12, 13].

Recent progress has also reshaped the translational landscape. Blood‐based screening has entered US regulated practice with FDA approval of a cfDNA‐based blood test for average‐risk adults aged 45 years or older, whereas ctDNA has established strong prognostic value and shown decision‐support utility in selected, stage‐specific settings; its broader treatment‐guiding utility, however, remains under prospective evaluation [14, 15, 16, 17]. In parallel, organoid platforms, spatial multiomics, and immune‐microenvironment studies have made it increasingly clear that pathway activity, stromal architecture, immune contexture, and host ecology must be interpreted together rather than as isolated layers of information [18, 19, 20, 21].

The present review therefore adopts a systems‐oriented and clinically translational structure. It first outlines epidemiological patterns and risk determinants, then examines signaling networks, tumor ecology, and microbiota‐related mechanisms that shape CRC evolution. It next reviews clinical presentation, screening, diagnosis, and molecular workup, followed by stage‐ and biomarker‐adapted management, translational models, and the clinical development of emerging therapies. This organization is intended to mirror the progression from population risk to tumor biology and, finally, to precision prevention and treatment. Table 1 summarizes the major practice‐shaping developments discussed in this review. Throughout the review, established standards are distinguished from practice‐changing developments in selected settings and from approaches that remain promising but investigational.

**TABLE 1:** Practice‐shaping developments in colorectal cancer.

Domain	Selected advance	Clinical significance	References
Epidemiology	Multinational analyses confirmed rising EOCRC incidence across diverse settings.	Supports cohort‐sensitive prevention, earlier symptom vigilance, and risk‐adapted screening rather than age‐only models.	[12, 13, 22]
Screening	FDA‐approved blood‐based cfDNA screening entered US clinical practice for average‐risk adults aged 45 years or older.	May improve participation among unscreened average‐risk adults; positive results require diagnostic colonoscopy, and precursor‐lesion sensitivity remains limited. It should be viewed as a participation‐enhancing option rather than a preventive equivalent to FIT or colonoscopy.	[14, 17, 23, 24, 25, 26]
Endoscopy	Expanded randomized evidence for AI‐assisted colonoscopy.	Functions as a quality amplifier that can improve adenoma and polyp detection when embedded in high‐quality endoscopy.	[27, 28, 29, 30, 31]
Liquid biopsy	Prospective cohorts and randomized trials clarified the prognostic value and clinical‐utility boundaries of postoperative ctDNA.	Provides strong postoperative recurrence‐risk stratification. Randomized evidence supports reduced chemotherapy use in selected patients with Stage II colon cancer, whereas routine Stage III escalation or de‐escalation and treatment initiated solely at molecular recurrence remain unvalidated.	[15, 32, 33, 34, 35]
Localized dMMR disease	Durable clinical complete responses enabling nonoperative management in selected locally advanced dMMR rectal cancer, together with high pathological response rates after neoadjuvant immunotherapy in resectable dMMR colon cancer.	Supports organ preservation in carefully selected rectal‐cancer patients treated at experienced centers with rigorous surveillance. In colon cancer, neoadjuvant immunotherapy is highly active but remains integrated with planned surgical resection rather than replacing surgery.	[36, 37, 38]
mCRC targeted therapy	KRAS G12C combinations, broader HER2‐directed strategies, and first‐line BRAF/EGFR‐directed intensification in BRAF V600E‐mutant mCRC.	Expands sequencing options and deepens the need for comprehensive molecular profiling in selected biomarker‐defined subsets.	[39, 40, 41, 42, 43, 44]

Abbreviations: ctDNA, circulating tumor DNA; dMMR, deficient mismatch repair; EOCRC, early‐onset colorectal cancer; mCRC, metastatic colorectal cancer; MRD, molecular residual disease; PD‐1, programmed cell death protein 1.

## 2 Epidemiology

CRC represents a major and evolving global health challenge, the epidemiological landscape of which reflects the interplay among demographic transition, socioeconomic development, and shifting exposure to environmental risk factors [1, 22, 45]. Over recent decades, the global burden of CRC has increased substantially in absolute terms, largely driven by population growth and aging [1, 2]. By contrast, age‐standardized incidence and mortality rates display marked regional heterogeneity, underscoring the influence of both biological and systemic determinants [45]. Understanding these dynamics is essential not only for informing prevention strategies and optimizing screening programs but also for linking population‐level patterns with underlying disease mechanisms.

### 2.1 Global Incidence and Mortality

CRC remains among the most commonly diagnosed cancers worldwide and a leading cause of cancer‐related death [1, 2]. Global estimates consistently rank CRC among the three most commonly diagnosed cancers and as the second leading cause of cancer death worldwide [46].

Notably, age‐standardized incidence and mortality rates have stabilized or declined in several high‐income countries, largely attributable to the implementation of screening programs and modification of key risk factors [11, 22]. However, the absolute number of CRC cases continues to rise, reflecting demographic shifts, particularly population aging. This apparent paradox highlights the importance of distinguishing between relative and absolute disease burden when evaluating epidemiological trends [47].

According to GLOBOCAN 2024, an estimated 2,041,007 new CRC cases and 917,895 related deaths occurred worldwide in 2024, accounting for approximately 9.9% of all newly diagnosed cancers and 9.4% of cancer deaths [48]. CRC therefore ranked among the most commonly diagnosed malignancies and remained the second leading cause of cancer mortality globally.

### 2.2 Geographic and Socioeconomic Disparities

Marked geographic variation in CRC incidence and mortality reflects differences in both environmental exposures and healthcare systems [22]. High‐income regions—including North America, Western Europe, Australia, and New Zealand—have historically exhibited the highest incidence rates, largely associated with “westernized” lifestyles characterized by high consumption of red and processed meats, sedentary behavior, and increased prevalence of obesity [49]. In contrast, many low‐ and middle‐income countries have traditionally exhibited lower incidence but are now experiencing rapid increases, coinciding with urbanization and lifestyle transitions [50]. These observations suggest that CRC risk is highly responsive to environmental and behavioral changes.

Socioeconomic disparities further shape disease outcomes. Countries with higher Human Development Index (HDI) generally achieve more favorable outcomes, reflecting broader access to screening, early diagnosis, and advanced treatment [49, 50]. Conversely, limited healthcare infrastructure and low screening coverage in resource‐constrained settings contribute to delayed diagnosis and increased mortality [45, 51]. Collectively, these disparities highlight CRC as a disease influenced not only by biology but also by health system capacity and policy.

### 2.3 Age and Sex Distribution

CRC predominantly affects older adults, with incidence increasing sharply after the age of 50 years, reflecting cumulative genetic alterations and prolonged exposure to environmental risk factors [22]. However, a notable and increasingly concerning trend is the rise in EOCRC, defined as CRC diagnosed before 50 years of age [13]. The threshold of 50 years is primarily an epidemiological convention rather than a discrete biological boundary, and risk increases along a continuum shaped by age, inherited susceptibility, and cumulative exposure history. In contrast to declining incidence in older populations, EOCRC rates have increased across multiple regions, suggesting the contribution of additional etiological factors beyond aging [12]. Observational studies have associated EOCRC risk with obesity, dietary patterns, antibiotic exposure, and microbiome perturbations. These factors are biologically plausible contributors through metabolic, inflammatory, and microbial pathways, but their causal effects, critical exposure windows, relative contributions, and precise mechanisms remain incompletely defined [10, 13].

Sex differences are also evident, with men generally exhibiting higher incidence and mortality than women. These differences likely reflect a combination of behavioral factors, hormonal influences, and biological variation. Notably, emerging evidence suggests sex‐specific differences in tumor location and molecular characteristics, indicating underlying heterogeneity that warrants further investigation [52, 53].

A particularly important recent finding is that a large multinational registry analysis reported rising CRC incidence in adults aged 25–49 years in 27 of 50 countries and territories, while rates in older adults were stable or falling in many of the same settings [12]. This divergence is consistent with birth‐cohort effects and suggests that early‐life and cumulative exposures may contribute to EOCRC risk, although the relative contribution and causal role of individual exposures remain uncertain [13, 22].

### 2.4 Temporal Trends

Temporal trends in CRC epidemiology reveal a divergence across both regions and age groups. In many high‐income countries, the implementation of screening programs—particularly colonoscopy and fecal‐based testing—has led to substantial reductions in incidence and mortality among older adults, primarily through early detection and removal of precancerous lesions [1, 11, 54].

By contrast, increasing incidence among younger individuals represents an emerging and unresolved challenge. The underlying drivers are likely multifactorial, involving dietary changes, increasing obesity prevalence, sedentary lifestyles, and alterations in gut microbiota composition. Although improved detection and heightened awareness may contribute, these factors alone are insufficient to fully explain the observed trends, suggesting additional, as yet unidentified mechanisms [12, 13, 22].

### 2.5 Screening Coverage and Its Impact on Epidemiology

Screening plays a central role in shaping CRC epidemiology by enabling both early detection and prevention [11]. Countries with well‐established, population‐based screening programs have achieved substantial reductions in CRC incidence and mortality. However, screening coverage varies widely across regions, with high uptake in some high‐income countries and persistently low participation in many low‐ and middle‐income settings [50]. Even within countries, disparities in participation across socioeconomic and demographic groups further exacerbate inequalities in disease burden [55, 56]. These observations underscore the importance of not only implementing screening programs but also ensuring equitable access and adherence.

Improving screening coverage and participation therefore represents one of the most effective and scalable strategies for reducing CRC burden worldwide.

### 2.6 Future Projections

Projections indicate that the global burden of CRC will continue to increase over the coming decades, with substantial rises in both incidence and mortality expected by 2040–2050 [1, 2, 47]. These increases are driven primarily by demographic changes, including population growth and aging, as well as continued exposure to modifiable risk factors.

Without effective intervention, CRC is likely to impose an increasing strain on healthcare systems, particularly in low‐ and middle‐income countries [57, 58]. These projections highlight the urgent need for integrated cancer‐control strategies that combine prevention, early detection, and equitable access to diagnosis and treatment.

### 2.7 Integrative Perspective

Overall, CRC epidemiology is characterized by a paradoxical pattern of declining rates in screened populations but an increasing global burden, particularly among younger individuals and in transitioning economies. These trends reflect the combined effects of demographic change, lifestyle transitions, and unequal access to healthcare [12, 22].

Importantly, epidemiological patterns are closely linked to underlying biological mechanisms, including metabolic dysfunction, inflammation, and microbiota alterations. This integrative perspective provides a conceptual bridge between population‐level observations and molecular pathogenesis, setting the stage for the subsequent discussion of CRC risk factors and etiological determinants [4, 10, 13]. To contextualize the foregoing epidemiological observations within the broader continuum of CRC development and care, Figure 1 presents an integrated systems‐level overview linking population risk, molecular dysregulation, tumor ecosystem dynamics, and adaptive precision management.

**FIGURE 1:**
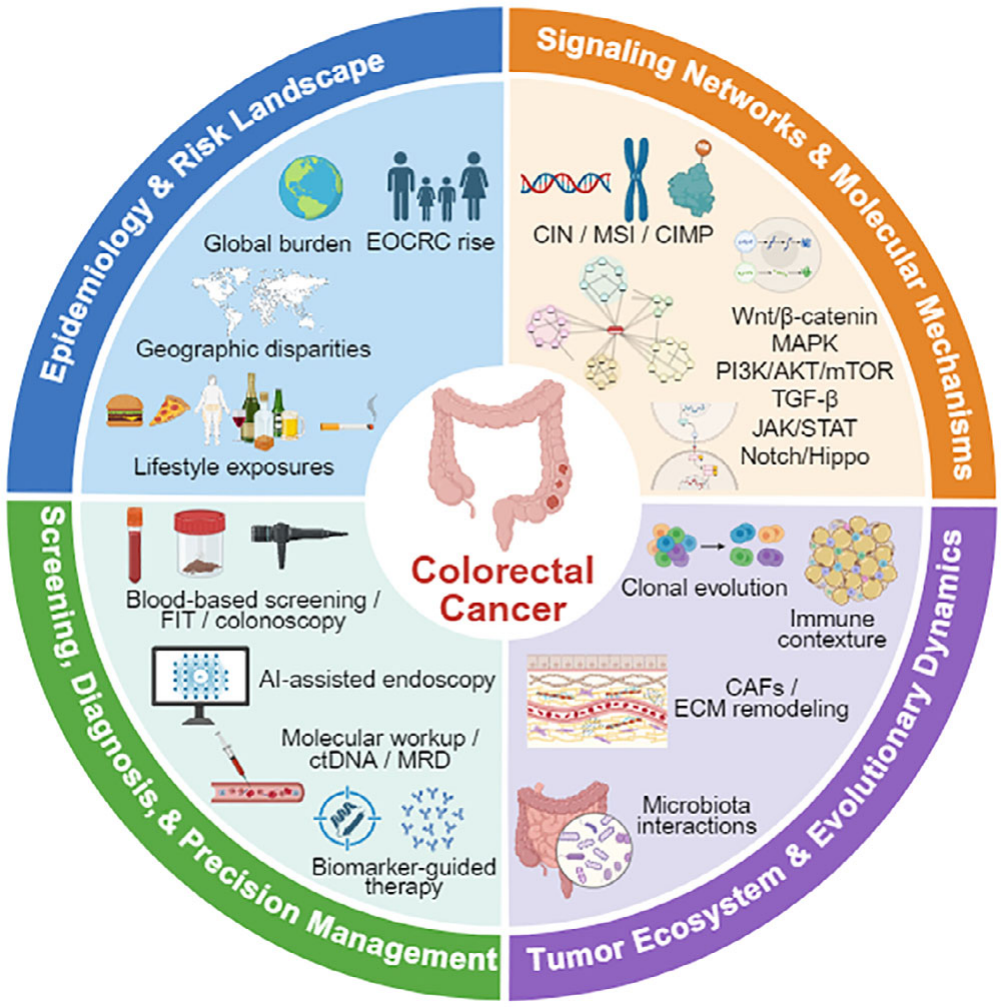
Colorectal cancer as a systems disease across the prevention‐to‐care continuum. This overview illustrates colorectal cancer (CRC) as a multiscale and adaptive disease shaped by the interaction of population‐level risk exposures, molecular signaling networks, tumor ecosystem dynamics, and clinically integrated management. The epidemiology and risk landscape includes global disease burden, the rise of early‐onset CRC, geographic and socioeconomic disparities, and modifiable lifestyle exposures. These determinants converge on interconnected molecular mechanisms, including genomic and epigenetic instability, dysregulation of Wnt/β‐catenin, MAPK, PI3K/AKT/mTOR, TGF‐β, and immune‐related signaling pathways. At the tumor level, CRC evolves within a structured ecosystem characterized by clonal diversification, immune contexture, stromal remodeling, and microbiota‐related interactions, which together influence heterogeneity, progression, and therapeutic resistance. Clinically, this systems framework connects screening, diagnosis, molecular workup, liquid biopsy, and biomarker‐guided treatment across the continuum from prevention and early detection to adaptive precision care. AI, artificial intelligence; AKT, protein kinase B; CAFs, cancer‐associated fibroblasts; CIMP, CpG island methylator phenotype; CIN, chromosomal instability; CRC, colorectal cancer; ctDNA, circulating tumor DNA; ECM, extracellular matrix; EOCRC, early‐onset colorectal cancer; FIT, fecal immunochemical test; JAK, Janus kinase; MAPK, mitogen‐activated protein kinase; MRD, molecular residual disease; MSI, microsatellite instability; mTOR, mechanistic target of rapamycin; PI3K, phosphoinositide 3‐kinase; STAT, signal transducer and activator of transcription; TGF‐β, transforming growth factor beta; Wnt, Wingless/Int‐1.

## 3 Risk Factors

The epidemiological landscape of CRC reflects the cumulative and interactive effects of multiple risk determinants spanning genetic, environmental, metabolic, and microbial domains. Although aging remains the strongest nonmodifiable risk factor, accumulating evidence indicates that modifiable exposures substantially contribute to CRC risk and may interact with inherited susceptibility, aging, inflammation, and host biology [13, 22].

Importantly, these factors do not operate in isolation but converge on a limited set of biological processes, including chronic inflammation, metabolic dysregulation, genomic instability, and remodeling of the tumor microenvironment (TME) [59]. This convergence provides a mechanistic framework linking population‐level risk exposures to molecular alterations and tumorigenesis [4, 59]. Understanding how these exposures are translated into signaling dysregulation is therefore central to prevention, risk stratification, and early detection.

### 3.1 Nonmodifiable Risk Factors

#### 3.1.1 Age

CRC incidence increases markedly with age, reflecting the progressive accumulation of somatic mutations and prolonged exposure to carcinogenic influences. However, the increasing incidence of EOCRC challenges this paradigm, demonstrating that clinically significant CRC can arise before older age and motivating investigation of life‐course environmental, metabolic, inflammatory, and microbiota‐related exposures [13, 22]. These observations indicate that chronological age alone is insufficient to capture risk, and that biological aging and exposure history must also be considered.

#### 3.1.2 Sex

Men exhibit higher incidence and mortality rates than women, a difference likely attributable to both behavioral and biological factors [52]. Hormonal influences, particularly the protective effects of estrogen, have been proposed to modulate inflammation, metabolism, and epithelial homeostasis. In addition, sex‐specific differences in immune responses and microbiota composition may contribute to differential susceptibility, although these mechanisms remain incompletely defined [53, 60].

#### 3.1.3 Genetic Predisposition and Family History

A positive family history of CRC confers a substantial increase in individual risk, and hereditary syndromes such as Lynch syndrome and familial adenomatous polyposis (FAP) provide important mechanistic insights into mismatch repair deficiency and Wnt signaling dysregulation [3, 61, 62, 63]. At the same time, most CRC remains sporadic, underscoring the importance of gene–environment interplay rather than purely inherited risk.

### 3.2 Lifestyle‐Related Risk Factors

#### 3.2.1 Diet

Dietary patterns represent one of the most influential and modifiable determinants of CRC risk. High consumption of red and processed meats is associated with increased CRC risk, whereas dietary patterns characterized by higher intakes of whole grains, fiber‐rich foods, and plant‐derived foods are generally associated with lower risk [64, 65].

Mechanistically, diet influences CRC development through multiple interconnected pathways. These include the generation of carcinogenic compounds such as N‐nitroso compounds, modulation of gut microbiota composition and function, and regulation of epithelial turnover and inflammatory signaling. These processes converge on key oncogenic pathways, linking environmental exposure directly to molecular dysregulation [4, 59, 66].

Notably, increasing consumption of ultraprocessed foods has emerged as an additional risk factor, potentially acting through metabolic disruption, chronic inflammation, and alterations in microbial ecology, although causal mechanisms remain to be fully elucidated [13, 67].

Recent work has strengthened the view that diet‐related carcinogenesis should be interpreted through patterns rather than isolated nutrients [65, 68]. In particular, obesity‐related and ultraprocessed food exposures are increasingly discussed as plausible contributors to EOCRC via insulin resistance, bile acid perturbation, systemic inflammation, and microbiome remodeling. Although causality remains difficult to prove, the convergence of metabolic and microbial evidence supports incorporating food processing intensity and life‐course metabolic health into CRC risk models [68].

#### 3.2.2 Alcohol Consumption

Alcohol consumption increases CRC risk in a dose‐dependent manner [69]. Ethanol metabolism produces acetaldehyde, a highly reactive metabolite that induces DNA damage, impairs DNA repair mechanisms, and promotes genomic instability [70, 71]. These effects may be amplified by oxidative stress and by interactions between alcohol exposure and folate‐related one‐carbon metabolism, although the magnitude and clinical relevance of these interactions vary across populations [72].

#### 3.2.3 Tobacco Smoking

Tobacco smoking contributes to CRC through exposure to carcinogens that induce DNA mutations and promote adenoma formation. Smoking has also been associated with selected molecular and epigenetic features of CRC, including differences in mutational and DNA‐methylation patterns, although these associations are heterogeneous and do not define a uniform smoking‐related CRC subtype [73, 74, 75].

#### 3.2.4 Physical Inactivity

Physical inactivity is associated with increased CRC risk, whereas higher habitual physical activity is associated with lower risk in prospective epidemiological studies. Potential mechanisms include improved insulin sensitivity, reduced adiposity and systemic inflammation, and modulation of immune and metabolic homeostasis. Because most incidence‐risk evidence remains observational, these mechanisms should be interpreted as biologically plausible rather than definitively causal [76, 77, 78].

### 3.3 Obesity, Metabolic Disorders, and Diabetes

Obesity represents a major and increasingly prevalent modifiable risk factor for CRC. General obesity and adverse body‐composition phenotypes, including visceral adiposity, have been linked to increased CRC risk and may influence clinical outcomes [79, 80, 81].

At the mechanistic level, obesity induces a chronic proinflammatory state characterized by elevated cytokines, adipokines, and insulin resistance. Hyperinsulinemia and activation of insulin‐like growth factor (IGF) signaling promote cellular proliferation and inhibit apoptosis, linking metabolic dysfunction to oncogenic pathway activation, particularly within the PI3K–AKT axis [13, 82].

Metabolic syndrome and Type 2 diabetes are associated with increased CRC risk, plausibly through hyperinsulinemia, chronic inflammation, altered adipokine signaling, and metabolic dysfunction [83]. Notably, early‐life obesity has been increasingly associated with EOCRC, suggesting that prolonged metabolic dysregulation may contribute to earlier disease development, although causality and critical exposure windows remain uncertain [84].

### 3.4 Chronic Inflammation and Inflammatory Bowel Disease

Chronic inflammation is a well‐established driver of colitis‐associated colorectal carcinogenesis and may also contribute to selected sporadic CRC contexts. Patients with inflammatory bowel disease (IBD) exhibit a substantially increased risk of CRC, particularly in the context of long‐standing and extensive disease [85, 86].

Persistent inflammation promotes continuous epithelial turnover, increases DNA damage, and generates a protumorigenic microenvironment characterized by cytokine signaling, immune cell infiltration, and oxidative stress. These processes activate key signaling pathways—including NF‐κB and STAT3—that directly interact with oncogenic networks and facilitate tumor initiation and progression [4].

### 3.5 Gut Microbiota and Microbial Dysbiosis

The gut microbiota has emerged as a plausible mediator linking environmental exposures, host metabolism, mucosal immunity, and CRC development. Dysbiosis has been implicated in tumorigenesis through several nonexclusive mechanisms, including production of genotoxic metabolites, induction of chronic inflammation, modulation of host immune responses, and disruption of epithelial integrity [59, 87]. Specific bacterial species—including *Fusobacterium nucleatum*, pks‐positive *Escherichia coli* and enterotoxigenic *Bacteroides fragilis*—have been associated with activation of oncogenic signaling pathways and promotion of tumor‐associated inflammation, whereas commensal microbes that generate short‐chain fatty acids such as butyrate may help maintain barrier function and suppress inflammation [88]. The microbiota also appears to influence therapeutic response, particularly in the context of immunotherapy, although many reported associations remain sensitive to confounding by diet, inflammation, transit time, and host metabolic status [89].

### 3.6 Risk Factors Specific to EOCRC

EOCRC appears to have a partially distinct but incompletely defined risk profile compared with CRC diagnosed at older ages. Although hereditary factors contribute to a subset of cases, the majority remain sporadic [13, 22, 90].

Emerging evidence implicates obesity, high intake of ultraprocessed foods, alcohol consumption, and antibiotic exposure as plausible contributors. Early‐life disruption of the gut microbiota may be particularly relevant through long‐term effects on immune development and metabolic regulation, although causality remains difficult to establish [91, 92, 93]. EOCRC is frequently diagnosed at more advanced stages, partly because symptoms are often under‐recognized and routine screening is absent in younger populations. These observations highlight a critical gap in current prevention strategies [94, 95].

Beyond heredity, current evidence has motivated a hypothesis‐generating multihit model in which early‐life or young‐adult obesity, altered microbial maturation, repeated antibiotic exposure, circadian disruption, and energy‐dense dietary patterns may interact in biologically susceptible individuals. The relative contribution and causal role of these exposures remain unresolved [13, 96]. The frequent advanced‐stage presentation of EOCRC is more directly associated with the absence of routine screening in younger adults, lower clinical suspicion, and delayed evaluation of warning symptoms, and should not be presented as proof of this exposure model.

### 3.7 Integrated Perspective on CRC Risk

CRC risk factors form an interconnected network rather than a series of independent variables. Genetic susceptibility establishes a baseline upon which environmental and lifestyle exposures act, converging on a limited set of biological processes that include inflammation, metabolic dysregulation, oxidative stress, barrier dysfunction, and microbial imbalance [1, 22].

These shared perturbations provide the mechanistic bridge between epidemiology and molecular pathogenesis and explain why clinically diverse exposures can funnel into a restricted set of tumor‐promoting processes. This convergence framework provides a useful basis for prevention, risk stratification, and interpretation of downstream signaling dysregulation [59, 88]. The mechanistic convergence by which heterogeneous risk exposures are translated into a restricted set of tumor‐promoting biological perturbations, culminating in colorectal tumor initiation, is delineated in Figure 2.

**FIGURE 2:**
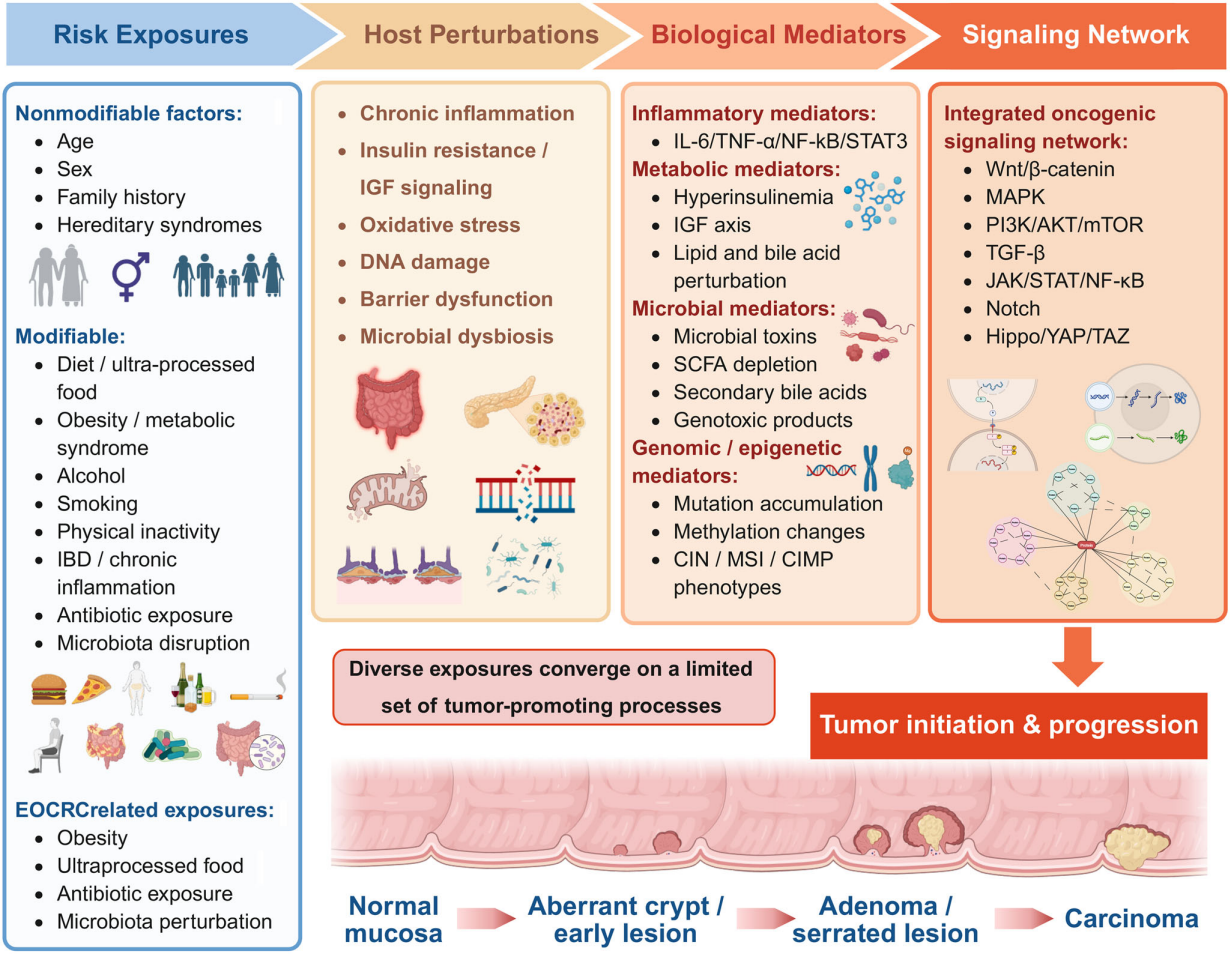
From risk exposures to colorectal tumor initiation: convergent pathways linking host, microbial, inflammatory, and molecular perturbations. Multiple nonmodifiable and modifiable risk factors, including aging, inherited susceptibility, diet, obesity, alcohol, smoking, physical inactivity, chronic inflammation, antibiotic exposure, and microbiota disruption, converge on a limited set of host perturbations such as chronic inflammation, metabolic dysfunction, oxidative stress, epithelial barrier injury, DNA damage, and microbial dysbiosis. These changes are mediated through inflammatory cytokines, metabolic signals, microbial products, and genomic or epigenetic alterations, which collectively activate interconnected oncogenic pathways including Wnt/β‐catenin, MAPK, PI3K/AKT/mTOR, TGF‐β, and JAK/STAT‐related signaling. Their cumulative effects promote epithelial dysregulation, precursor‐lesion development, and colorectal tumor initiation. AKT, protein kinase B; CIMP, CpG island methylator phenotype; CIN, chromosomal instability; CRC, colorectal cancer; EOCRC, early‐onset colorectal cancer; IBD, inflammatory bowel disease; IGF, insulin‐like growth factor; IL‐6, interleukin‐6; JAK, Janus kinase; MAPK, mitogen‐activated protein kinase; MSI, microsatellite instability; mTOR, mechanistic target of rapamycin; NF‐κB, nuclear factor kappa B; PI3K, phosphoinositide 3‐kinase; SCFAs, short‐chain fatty acids; STAT, signal transducer and activator of transcription; STAT3, signal transducer and activator of transcription 3; TAZ, transcriptional coactivator with PDZ‐binding motif; TGF‐β, transforming growth factor beta; TNF‐α, tumor necrosis factor alpha; Wnt, Wingless/Int‐1; YAP, yes‐associated protein.

## 4 Signaling Pathways and Molecular Mechanisms

CRC arises through a complex and dynamic process driven by the accumulation of genetic and epigenetic alterations within a permissive and evolving microenvironment. CRC is increasingly understood not as a disease governed by linear oncogenic pathways, but as a network‐driven system in which multiple signaling cascades operate in parallel and interact extensively [4, 8].

These signaling networks integrate tumor‐intrinsic alterations with extrinsic inputs derived from the TME, thereby regulating tumor initiation, clonal expansion, invasion, metastasis, immune evasion, and therapeutic resistance. At the genomic level, CRC develops through partially overlapping molecular routes characterized by chromosomal instability (CIN), microsatellite instability (MSI), and CpG island methylator phenotype (CIMP)—which collectively generate substantial intertumoral variability [7].

Concurrently, intratumoral heterogeneity emerges through ongoing clonal evolution and selective pressures within the tumor ecosystem, resulting in the coexistence of multiple subclonal populations with distinct molecular and phenotypic characteristics. Advances in single‐cell and spatially resolved technologies have further revealed that this heterogeneity is highly structured and context‐dependent, shaped by both intrinsic signaling states and microenvironmental cues [9, 97].

These insights have led to integrative classification frameworks, such as the consensus molecular subtype (CMS) system, which capture CRC heterogeneity by incorporating signaling activity, immune contexture, metabolic reprogramming, and stromal features. Collectively, these developments support a paradigm shift toward viewing CRC as a systems‐level disease governed by dynamic signaling networks and ecological interactions [98, 99].

### 4.1 Molecular Evolution

CRC evolution is best understood as a dynamic and branching process driven by genomic instability and shaped by microenvironmental selection pressures. Rather than following a strictly linear trajectory, tumor progression involves continuous clonal diversification, with multiple subclones emerging, competing, and adapting over time [8, 100, 101].

Distinct evolutionary routes—including the conventional adenoma–carcinoma sequence and the serrated pathway—are defined by characteristic mutational and epigenetic landscapes. These trajectories are associated with partially overlapping molecular features, including CIN, MSI, and CIMP, which promote genomic or epigenetic diversity through distinct mechanisms [5, 102].

Importantly, these processes generate extensive heterogeneity that underlies differences in tumor behavior, metastatic potential, and therapeutic response. This evolutionary perspective extends classical stepwise models and highlights the need to consider CRC as an adaptive system undergoing continuous selection [99].

### 4.2 Consensus Molecular Subtypes and Tumor Heterogeneity

The CMS framework provides an integrative model that captures CRC heterogeneity across genomic, transcriptomic, and microenvironmental dimensions. By stratifying tumors into four biologically distinct subtypes—CMS1 (MSI immune), CMS2 (canonical), CMS3 (metabolic), and CMS4 (mesenchymal)—this system links molecular features with biological behavior and prognosis, whereas reported associations with treatment response remain insufficiently prospective and reproducible for routine clinical decision‐making [8, 98, 103].

Importantly, CMS classification extends beyond tumor‐intrinsic signaling to incorporate immune and stromal components, reflecting the critical role of the TME in shaping disease phenotypes. CMS1 tumors are characterized by high mutational burden and immune activation, CMS2 by canonical Wnt/MYC signaling, CMS3 by metabolic reprogramming, and CMS4 by stromal infiltration and TGF‐β activation [20].

However, intratumoral heterogeneity frequently results in the coexistence of multiple CMS‐like states within a single tumor, complicating clinical application and contributing to therapeutic resistance. Thus, while CMS provides a valuable framework for patient stratification, its limitations underscore the need for more dynamic and spatially resolved classification systems [99, 104].

A major conceptual advance since the original CMS framework has been the increasing research use of single‐cell and spatially resolved profiling to dissect structured intratumor ecology. Recent CRC studies show that canonical, immune, stromal, and metabolic states can coexist within the same lesion and are distributed in anatomically distinct niches, especially at the invasive front and in immune‐excluded stromal compartments. Accordingly, CMS should be regarded as a biologically and prognostically informative framework rather than a stand‐alone predictive biomarker for routine treatment selection, particularly given intratumoral heterogeneity, stromal contributions, assay dependence, and the absence of prospectively validated therapeutic algorithms [105].

### 4.3 Canonical Oncogenic Signaling Pathways

The initiation and progression of CRC are governed by dysregulation of multiple canonical signaling pathways that operate as an interconnected network rather than as isolated cascades. Core oncogenic axes—including Wnt/β‐catenin, MAPK, PI3K/AKT/mTOR, and TGF‐β signaling—regulate complementary aspects of tumor biology such as stem cell maintenance, proliferation, metabolic adaptation, epithelial–mesenchymal transition, and immune modulation [106, 107, 108].

A defining feature of these pathways is extensive crosstalk and feedback regulation. This architecture allows tumor cells to preserve signaling output, adapt to environmental stress, and rewire pathway dependence under therapeutic pressure [109].

From a translational standpoint, this network architecture helps explain why single‐pathway inhibition is often insufficient and why durable benefit increasingly depends on biomarker‐guided combinations and context‐specific targeting strategies [110, 111].

#### 4.3.1 Wnt/β‐Catenin Pathway

The Wnt/β‐catenin pathway represents the foundational driver of colorectal tumorigenesis and is dysregulated in the majority of CRC cases. Constitutive activation—most commonly through APC loss—leads to stabilization and nuclear accumulation of β‐catenin, which drives transcriptional programs governing proliferation, stemness, and metabolic adaptation [112, 113, 114].

Beyond tumor initiation, Wnt signaling sustains plastic stem‐like tumor‐cell states and contributes to tumor propagation, metastatic competence, and therapeutic resistance. Notably, its activity is spatially heterogeneous and often enriched at the invasive front, where it integrates with TME‐derived cues to promote epithelial–mesenchymal plasticity [115].

Importantly, Wnt signaling exhibits extensive crosstalk with PI3K/AKT, Notch, Hippo, and inflammatory pathways such as NF‐κB and JAK/STAT, linking stemness, metabolism, and immune modulation. Despite its central role, therapeutic targeting remains challenging due to its essential function in normal tissue homeostasis, underscoring the need for context‐dependent strategies [113, 114].

#### 4.3.2 MAPK Pathway

The MAPK pathway is a central regulator of proliferation, differentiation, and survival in CRC. Activating mutations in KRAS or BRAF drive constitutive signaling independent of receptor activation, promoting tumor growth and influencing therapeutic response [109, 116, 117].

A key feature of MAPK signaling is its adaptability. Targeted inhibition frequently induces compensatory feedback activation, including upregulation of upstream receptors or activation of parallel pathways such as PI3K/AKT. In addition, intratumoral heterogeneity enables expansion of resistant subclones, contributing to both primary and acquired resistance [110]. These properties support clinically validated vertical pathway blockade in selected contexts, such as combined BRAF and EGFR inhibition, while combinations involving MAPK and PI3K inhibition remain predominantly investigational because of toxicity, pathway redundancy, and limited therapeutic windows.

#### 4.3.3 PI3K/AKT/mTOR Pathway

The PI3K/AKT/mTOR pathway functions as a central metabolic and survival axis, integrating signals from growth factors, nutrients, and cellular stress. Aberrant activation—through PIK3CA mutations, PTEN loss, or receptor amplification—promotes proliferation, survival, and metabolic reprogramming [118, 119].

This pathway supports anabolic metabolism, including glycolysis, lipid synthesis, and protein production, enabling tumor cells to adapt to hypoxic and nutrient‐limited environments. Extensive crosstalk with MAPK signaling further enhances adaptability and contributes to therapeutic resistance [120].

Clinical targeting has been limited by feedback activation and toxicity, highlighting the need for biomarker‐driven and combination‐based approaches [110].

#### 4.3.4 TGF‐β/SMAD Pathway

The TGF‐β pathway exhibits a context‐dependent role in CRC, functioning as a tumor suppressor in early stages and a tumor promoter in advanced disease. During tumor progression, loss of growth‐inhibitory responses enables TGF‐β signaling to drive epithelial–mesenchymal transition, invasion, metastasis, and immune evasion [107, 121, 122].

Beyond tumor cells, TGF‐β plays a central role in shaping the TME by promoting fibroblast activation, extracellular matrix remodeling, and immunosuppression. These effects are particularly prominent in mesenchymal‐like, fibroblast‐rich states associated with poor prognosis [123].

Therapeutic targeting remains challenging due to its dual functions, but combination strategies with immunotherapy are under active investigation [124, 125].

### 4.4 Additional Pathways Involved in Progression and Metastasis

Beyond canonical oncogenic pathways, CRC progression and metastasis are driven by additional signaling networks that regulate cellular plasticity, inflammatory signaling, metabolic adaptation, and interactions with the TME. These pathways—including Notch, JAK/STAT, Hedgehog, Hippo/YAP/TAZ, and AMPK—function as context‐dependent modulators of tumor evolution, particularly in advanced disease characterized by increased heterogeneity and therapeutic pressure [106, 126, 127, 128, 129].

Rather than acting independently, these pathways are embedded within an interconnected signaling network with extensive crosstalk and feedback regulation. For example, Notch and Wnt cooperate to maintain stem‐like tumor‐cell states, JAK/STAT integrates inflammatory cues into proliferative and immunosuppressive programs, and Hippo/YAP/TAZ converges with TGF‐β signaling to drive epithelial–mesenchymal plasticity and metastatic competence. In parallel, AMPK links metabolic stress to survival signaling, enabling tumor cells to adapt to fluctuating energy conditions. Importantly, these pathways are dynamically shaped by TME‐derived inputs, including stromal interactions, immune infiltration, hypoxia, and microbiota‐derived signals. This network‐level integration allows tumor cells to rewire signaling dependencies under environmental and therapeutic stress, thereby promoting tumor heterogeneity, metastatic dissemination, and resistance to treatment [130].

#### 4.4.1 Notch Signaling

Notch signaling regulates cell fate determination and epithelial differentiation in the intestine and is tightly coordinated with Wnt signaling under physiological conditions [131, 132].

In CRC, aberrant activation disrupts this balance, promoting expansion of stem‐like tumor cells while inhibiting differentiation, particularly of secretory lineages. Mechanistically, ligand binding induces proteolytic cleavage and release of the Notch intracellular domain, which activates transcriptional programs sustaining proliferation and stemness. This activity cooperates with Wnt signaling to maintain stemness‐associated programs and supports tumor maintenance and recurrence. Beyond tumor initiation, Notch promotes epithelial–mesenchymal transition and metastatic progression through interaction with TGF‐β signaling. It also enhances survival through crosstalk with PI3K/AKT signaling, contributing to resistance under therapeutic stress [129, 133, 134].

Notch further modulates the TME by influencing fibroblast activation, angiogenic programs, and immune cell function, thereby establishing a protumorigenic and immunosuppressive environment. Although Notch signaling remains therapeutically attractive, clinical application is limited by toxicity, necessitating more selective and context‐dependent approaches [129, 133, 134].

#### 4.4.2 JAK/STAT Signaling

The JAK/STAT pathway links chronic inflammation to tumor progression and immune evasion, functioning as a key interface between environmental stimuli and tumor signaling. Cytokine‐driven activation of STAT3 induces transcriptional programs that promote proliferation, survival, angiogenesis, and immune modulation. Persistent STAT3 activation is associated with aggressive disease and poor outcomes [20, 126, 135].

In the TME, JAK/STAT signaling drives immunosuppression by promoting expansion of MDSCs, Tregs, and M2‐like macrophages while inhibiting cytotoxic immune responses. It can also participate in feed‐forward interactions with NF‐κB signaling, sustaining chronic inflammation and tumor‐promoting signaling [136, 137].

Extensive crosstalk with MAPK and PI3K pathways enables compensatory signaling and contributes to resistance. In addition, JAK/STAT signaling promotes epithelial–mesenchymal transition and tumor‐initiating cell states, integrating inflammation with tumor plasticity. Therapeutically, targeting this pathway remains challenging due to systemic toxicity and network redundancy, but combination strategies with immunotherapy are under active investigation [138, 139].

#### 4.4.3 Hedgehog Signaling

Hedgehog signaling in CRC appears to function largely through tumor–stroma interactions rather than through uniform direct activation in tumor cells. Tumor‐derived ligands activate stromal cells, leading to fibroblast reprogramming, extracellular matrix remodeling, and angiogenesis, thereby creating a supportive tumor niche [140, 141, 142].

This stromal activation may foster a fibrotic and immunosuppressive microenvironment associated with tumor progression and poor prognosis. Hedgehog signaling also interacts with TGF‐β, Wnt, and PI3K pathways, integrating stromal and tumor‐intrinsic signaling. Despite its biological importance, clinical targeting has shown limited efficacy, likely due to pathway redundancy and context‐dependent effects. Current approaches focus on combinatorial targeting strategies [130].

#### 4.4.4 Hippo/YAP/TAZ Signaling

The Hippo pathway regulates tissue homeostasis through control of YAP/TAZ activity, which, when activated, drives transcriptional programs promoting proliferation, survival, and stemness. In CRC, YAP/TAZ activation is associated with tumor progression, metastasis, and poor prognosis [127, 143]. A defining feature of Hippo signaling is its integration with other pathways. YAP/TAZ interact with β‐catenin to enhance Wnt signaling and cooperate with TGF‐β to promote epithelial–mesenchymal transition, particularly in aggressive subtypes. Crosstalk with PI3K signaling further supports survival under stress conditions [144].

Hippo signaling is also highly responsive to mechanical cues such as extracellular matrix stiffness, linking biomechanical changes in the TME to oncogenic signaling. It contributes to fibroblast activation, angiogenesis, and immune modulation, further shaping tumor progression [20, 145]. Although direct targeting remains challenging, disrupting YAP–TEAD interactions and combination therapies represent promising strategies [146, 147].

#### 4.4.5 AMPK and Metabolic Regulation

AMPK functions as a central metabolic sensor that regulates energy homeostasis and exhibits context‐dependent roles in CRC. Under metabolic stress, AMPK activation promotes catabolic processes and suppresses mTOR signaling, exerting tumor‐suppressive effects. However, in established tumors, it can support survival under hypoxia and nutrient deprivation [148, 149, 150].

AMPK‐driven metabolic reprogramming enables tumor cells to adapt to energy stress, linking metabolic conditions such as obesity and diabetes with tumor biology. AMPK interacts with PI3K/AKT/mTOR signaling and may influence Wnt‐associated and stemness programs, although the direction and functional consequences of these interactions are highly context‐dependent [118]. Beyond tumor cells, AMPK affects TME interactions by modulating immune metabolism and responses to hypoxia and oxidative stress. Therapeutically, metabolic targeting strategies—including AMPK‐related approaches and metformin‐based interventions—are under investigation, although their context‐dependent effects require careful consideration [151, 152, 153].

Collectively, these additional pathways form a dynamic and adaptive signaling network that integrates oncogenic signaling with metabolic and microenvironmental inputs. Through extensive crosstalk and feedback regulation, they enable tumor cells to reprogram signaling dependencies in response to environmental stress and therapeutic intervention [106, 108, 154]. This network‐level plasticity underlies tumor heterogeneity, epithelial–mesenchymal transition, metastatic dissemination, and resistance to chemotherapy, targeted therapy, and immunotherapy. Importantly, incorporation of TME‐derived signals further amplifies these effects, reinforcing a protumorigenic and immunosuppressive niche [20]. From a translational perspective, this systems‐level organization highlights the limitations of single‐pathway targeting and supports the rational development of combinatorial and context‐specific therapeutic strategies [128].

### 4.5 Tumor Microenvironment, Immune Contexture, and Immune Escape

The TME is a central determinant of CRC progression, metastasis, and therapeutic response, functioning as a dynamic and heterogeneous ecosystem composed of immune cells, fibroblasts, endothelial cells, extracellular matrix, soluble factors, and microbiota‐derived metabolites. Rather than acting as a passive scaffold, it actively regulates tumor evolution through bidirectional interactions with cancer cells, influencing proliferation, invasion, angiogenesis, immune surveillance, and therapy resistance [9, 20, 155].

These interactions are spatially and temporally organized, with distinct niches—such as the invasive margin, hypoxic regions, and perivascular areas—often exhibiting unique cellular compositions and signaling activities that contribute to functional heterogeneity. CRC behavior therefore cannot be fully explained by tumor‐intrinsic alterations alone but must be understood within the context of the immune landscape, or immune contexture, which encompasses immune cell density, localization, activation state, and interactions with stromal and tumor components [156].

Immune contexture has direct clinical relevance. The Immunoscore can provide prognostic information beyond conventional clinicopathological staging in selected settings, but it complements rather than replaces the TNM system. CRC exhibits a spectrum of immune phenotypes, ranging from inflamed “hot” tumors with abundant cytotoxic T‐cell infiltration to immune‐excluded and immune‐desert tumors with limited immune engagement. These phenotypes are governed by tumor‐intrinsic signaling, stromal barriers, cytokine networks, and microbiota‐derived influences, and they critically determine therapeutic responsiveness, particularly to immunotherapy [157, 158, 159].

#### 4.5.1 MSI/dMMR and Neoantigen Burden

MSI is a key determinant of immune activation in CRC and results from deficiency in DNA mismatch repair, leading to accumulation of insertion–deletion mutations and a high tumor mutational burden. This generates abundant neoantigens that are presented to the immune system, driving strong cytotoxic T‐cell infiltration and activation of immune checkpoint pathways [20, 160].

MSI‐high tumors therefore exhibit a “hot” immune phenotype characterized by CD8^+^ T‐cell infiltration and upregulation of PD‐1, PD‐L1, and CTLA‐4, which underlies their sensitivity to immune checkpoint blockade. However, adaptive resistance can still occur through mechanisms such as impaired antigen presentation and immunosuppressive cytokine signaling [161].

In contrast, MSS tumors typically display low neoantigen burden, reduced immune infiltration, and poor responsiveness to immunotherapy. Importantly, MSI status alone does not fully capture immune complexity, as factors such as neoantigen quality, clonality, and stromal barriers also influence immune engagement. Subsets of MSS tumors with increased immune infiltration may retain limited or context‐dependent responsiveness, highlighting the need for refined immune stratification [162].

#### 4.5.2 Tumor‐Infiltrating Lymphocytes and Immune Contexture

The density, composition, and spatial distribution of tumor‐infiltrating lymphocytes are central determinants of CRC prognosis and treatment response. High infiltration of cytotoxic and memory T cells is associated with improved survival and effective immune surveillance, whereas low or spatially restricted infiltration is linked to poor outcomes [155, 157].

Beyond quantity, functional state is critical. T cells may range from fully active to exhausted phenotypes characterized by expression of inhibitory receptors. A TIL‐high phenotype has been shown to predict favorable prognosis independently of MSI status, including in subsets of MSS tumors [163].

The immune landscape is dynamic and influenced by cytokines, stromal barriers, metabolic competition, and microbiota‐derived signals. Interactions between TILs and other TME components can either support or suppress antitumor immunity, underscoring the complexity of immune regulation [155, 156, 157].

#### 4.5.3 Immune Checkpoint Pathways and Immune Evasion

CRC cells exploit immune checkpoint pathways to suppress T‐cell activity and evade immune surveillance. The PD‐1/PD‐L1 and CTLA‐4 axes are central mediators of T‐cell inhibition, leading to reduced effector function and T‐cell exhaustion [164].

While immune checkpoint blockade is highly effective in MSI‐high tumors, most CRCs exhibit resistance due to additional immune escape mechanisms, including defective antigen presentation, immunosuppressive cytokine networks, and recruitment of regulatory immune cells [165, 166]. Stromal barriers further restrict immune cell infiltration, contributing to immune‐excluded phenotypes.

Importantly, immune evasion is actively regulated by oncogenic pathways such as Wnt/β‐catenin and TGF‐β signaling, which suppress immune activation and promote immune exclusion. These findings highlight the integration of oncogenic signaling and immune regulation [167].

Overcoming immune resistance requires combinatorial strategies that integrate checkpoint blockade with therapies targeting stromal, metabolic, and signaling pathways to overcome defined mechanisms of immune exclusion and improve T‐cell infiltration or function; however, reliable conversion of MSS CRC into a broadly immunotherapy‐responsive phenotype has not been demonstrated [168].

Spatial profiling has further clarified that immune contexture is not merely a matter of total lymphocyte density. Instead, CRC tissues contain spatially segregated cytotoxic, exhausted, stromal‐barrier, and suppressive myeloid niches, and these architectures can differ substantially even among tumors with similar mutational classes. This helps explain why MSI status, although highly informative, does not by itself capture all clinically relevant determinants of response to immunotherapy [169, 170].

#### 4.5.4 Stromal Remodeling and CAF‐Mediated Progression

Stromal components, particularly CAFs, play a central role in CRC progression by orchestrating tumor–stroma interactions. CAFs promote tumor growth, invasion, and angiogenesis through secretion of growth factors, cytokines, and extracellular matrix components. ECM remodeling increases tissue stiffness and forms physical barriers that limit immune infiltration and drug delivery, contributing to immune exclusion and therapeutic resistance [20, 171]. CAFs also exert immunosuppressive effects by recruiting regulatory immune cells and inhibiting cytotoxic responses. These processes are closely linked to signaling pathways such as TGF‐β, Wnt, Hedgehog, and Hippo, particularly in mesenchymal subtypes associated with poor prognosis. CAF‐mediated remodeling also supports resistance by protecting tumor cells from therapeutic stress [142, 172]. Targeting selected CAF states or functions represents a biologically plausible but still investigational strategy, although their heterogeneity necessitates context‐specific approaches to avoid unintended effects [173].

The TME in CRC represents a dynamic and integrated system that coordinates immune, stromal, and signaling networks to regulate tumor progression and therapeutic response. Immune contexture, MSI status, and TIL infiltration provide complementary insights into prognosis and treatment stratification, while stromal remodeling imposes critical barriers to effective therapy [20].

Importantly, the TME evolves under tumor progression and therapeutic pressure, particularly in MSS CRC, where immune resistance remains a major challenge. These findings underscore the need for integrative therapeutic strategies targeting both tumor‐intrinsic signaling and microenvironmental regulation to achieve durable clinical benefit [174].

### 4.6 Gut Microbiota–Signaling Interactions

The gut microbiota has emerged as a potential regulator of CRC biology at the interface of environmental exposure, host metabolism, mucosal immunity, and signaling networks. As a dynamic ecosystem, it can influence epithelial integrity, inflammatory tone, and key signaling pathways that govern proliferation and survival [59, 175, 176].

Microbiota‐derived signals may contribute not only to early tumor initiation through DNA damage, genomic instability, and chronic inflammation, but also to later tumor evolution and therapeutic response. These effects intersect with Wnt/β‐catenin, NF‐κB, JAK/STAT, and PI3K/AKT signaling, as well as with metabolic and immune programs in the TME. Microbiota effects are highly context‐dependent, spatially organized, and vulnerable to confounding by diet, transit time, inflammation, and host metabolic state [89, 177].

#### 4.6.1 Microbiota Dysbiosis and CRC Initiation

Dysbiosis has been proposed as an early contributor to colorectal carcinogenesis, although whether it is a cause, consequence, or reinforcing component of tumor development varies across experimental and clinical contexts. CRC‐associated microbiota are characterized by enrichment of protumorigenic bacteria and depletion of beneficial commensals, resulting in impaired epithelial barrier function and increased mucosal permeability [178].

This disruption facilitates exposure of epithelial cells to microbial toxins and PAMPs, inducing DNA damage, genomic instability, and activation of innate immune signaling pathways such as NF‐κB and inflammasome pathways. In parallel, dysbiosis drives chronic low‐grade inflammation, with sustained cytokine production activating oncogenic signaling cascades including JAK/STAT and NF‐κB, thereby promoting proliferation and inhibiting apoptosis [179, 180].

Dysbiotic microbiota also alter metabolic outputs, including short‐chain fatty acids, bile acids, and polyamines, which influence differentiation, oxidative stress, and DNA repair. These effects are strongly influenced by diet, antibiotics, and metabolic status, providing a mechanistic link between environmental risk factors and tumor initiation. Experimental evidence from animal models and microbiota‐transfer studies supports a functional contribution of dysbiosis to CRC initiation, although causal assignments for individual taxa remain under active investigation [59, 87].

#### 4.6.2 Microbial Regulation of Oncogenic Signaling Pathways

One mechanism through which selected microbial communities may contribute to CRC is the modulation of host signaling pathways, genotoxic stress, epithelial integrity, and inflammatory responses. These effects are context‐dependent and should not be interpreted as evidence that the microbiota functions as a uniform upstream driver across all CRC subtypes [59, 176].

Specific bacteria can directly activate signaling cascades. For example, *Fusobacterium nucleatum* activates Wnt/β‐catenin signaling through interaction with epithelial adhesion molecules and stimulates TLR‐mediated NF‐κB activation, promoting inflammation and tumor growth. Similarly, enterotoxigenic *Bacteroides fragilis* induces STAT3 and NF‐κB signaling through toxin‐mediated epithelial disruption, driving inflammatory and proliferative responses [181, 182].

In addition, certain *Escherichia coli* strains produce genotoxins that induce DNA damage and CIN, complementing signaling activation with direct genomic effects [183, 184].

Beyond these examples, microbiota‐derived signals influence broader signaling networks—including PI3K/AKT, MAPK, and epigenetic regulation—through metabolites and host–microbe interactions. These effects are context‐dependent and contribute to interpatient heterogeneity in tumor progression and treatment response [176, 177].

#### 4.6.3 Microbiota and Immune Modulation in CRC

The gut microbiota may modulate immune contexture by influencing innate sensing, antigen presentation, dendritic‐cell function, and T‐cell priming [59, 185, 186]. Selected microbial configurations have been associated with enhanced cytotoxic immunity, whereas others have been associated with regulatory or myeloid‐dominant immune states. These associations may contribute to inflamed or immune‐excluded phenotypes but do not independently determine whether a tumor is immunologically “hot” or “cold.” Reported relationships with immunotherapy response remain vulnerable to confounding by diet, medication use, inflammation, intestinal transit time, geographic background, and host metabolic status [88].

#### 4.6.4 Microbial Metabolites and Metabolic Signaling

Microbial metabolites provide a key link between microbiota and host metabolic signaling in CRC. Short‐chain fatty acids, particularly butyrate, are generally considered to exert antitumor effects by regulating epigenetic modifications, promoting differentiation, and suppressing proliferation and inflammation, although some responses appear context‐dependent [187, 188, 189]. In contrast, other metabolites—such as secondary bile acids and polyamines—can promote tumorigenesis by inducing oxidative stress, DNA damage, and proliferative signaling. These metabolites also interact with oncogenic pathways and influence epigenetic regulation [190, 191, 192]. Microbial metabolites function both locally and systemically, contributing to metabolic reprogramming and adaptation to hypoxic and nutrient‐limited conditions. Their effects are highly dependent on diet, microbial composition, and host metabolism.

#### 4.6.5 Therapeutic Implications and Microbiota‐Targeted Strategies

Recognition of the microbiota as a regulator of CRC biology has important translational implications. Strategies including probiotics, dietary modulation, and fecal microbiota transplantation aim to restore microbial balance and enhance antitumor immunity [87, 193]. Microbiota composition influences therapeutic response, particularly to immunotherapy, by modulating antigen presentation, immune activation, and checkpoint signaling. It also affects chemotherapy and targeted therapy through drug metabolism and inflammatory regulation. However, clinical translation is challenged by interindividual variability and context‐dependent effects. Future efforts should focus on identifying robust microbial biomarkers and developing personalized microbiota‐based interventions. Combination strategies integrating microbiota modulation with immunotherapy and targeted therapy represent a promising approach to overcoming resistance and improving outcomes.

Recent microbiome scholarship has also introduced a note of methodological caution. A 2024 synthesis highlighted that transit time, inflammation markers, and body mass index can confound or even dominate apparent microbiota–CRC associations if quantitative profiling and covariate control are insufficient. Thus, future microbiota‐based biomarker development must move beyond descriptive taxonomic enrichment toward functionally validated, confounder‐aware models [89, 194].

### 4.7 Translational Implications of Signaling Pathway Dysregulation

Dysregulation of signaling pathways in CRC provides the conceptual foundation for molecular classification, therapeutic stratification, and precision oncology. In practice, the most clinically useful translation of this biology lies in identifying which pathway states and tumor ecosystem features define actionable vulnerabilities, prognostic subsets, and likely resistance mechanisms [7, 109, 110].

Pathway activity is not purely tumor‐intrinsic. It is shaped by immune infiltration, stromal remodeling, metabolic constraints, and microbiota‐derived inputs, which together influence how individual tumors behave and respond to treatment [195].

This section therefore focuses on three translational consequences of signaling dysregulation: biologically informed stratification, rational targeting of pathway‐defined subsets, and the need for combination strategies that anticipate adaptive resistance.

#### 4.7.1 Molecular Stratification and Biomarker‐Driven Classification

Advances in genomic and multiomics profiling have enabled identification of key molecular alterations that serve as clinically actionable biomarkers for patient stratification [196]. Extended RAS testing is required before anti‐EGFR therapy because activating KRAS or NRAS mutations predict lack of benefit. BRAF V600E identifies an aggressive, poor‐prognosis molecular subgroup for which BRAF/EGFR‐directed combination therapy is clinically relevant. MSI/dMMR status guides immunotherapy and also informs Lynch syndrome screening, whereas HER2 amplification or overexpression identifies another actionable subset in appropriately selected advanced disease. Additional alterations, including rare fusions, PIK3CA mutations, and selected DNA repair defects, further expand the landscape of biomarker‐guided therapy [16, 197].

Beyond single‐gene markers, CMS classification provides a systems‐level framework for biological interpretation, prognostic research, and clinical‐trial stratification, but it is not currently supported as a routine stand‐alone tool for treatment selection. ctDNA can support longitudinal molecular monitoring in selected clinical settings, whereas single‐cell profiling remains primarily a research tool for resolving heterogeneity and resistance mechanisms. Neither should be presented as a universally established adaptive‐treatment platform [8, 99].

#### 4.7.2 Targeted Therapies Based on Oncogenic Signaling Pathways

Targeted therapies directed against dysregulated signaling pathways have reshaped CRC treatment, but their current role is highly context‐dependent. Inhibition of the EGFR/MAPK axis with monoclonal antibodies is effective in RAS wild‐type tumors, whereas BRAF V600E‐mutant CRC requires combination targeting because pathway crosstalk rapidly limits single‐agent activity. HER2 amplification defines another actionable subset, while direct targeting of PI3K/AKT/mTOR has so far been constrained by pathway redundancy and toxicity. Taken together, these experiences show that pathway‐directed treatment is most successful when deployed in molecularly restricted populations rather than as a generalized strategy [198].

Within this framework, PI3K/AKT/mTOR‐targeted therapy remains limited by toxicity and pathway redundancy, whereas HER2‐directed therapy has clearer relevance in selected refractory, biomarker‐positive disease. Across all of these settings, compensatory signaling, clonal evolution, and TME influences remain major constraints, reinforcing the need for network‐level rather than single‐node therapeutic strategies [199].

#### 4.7.3 Immunotherapy and Immune‐Related Signaling Pathways

Immunotherapy has transformed treatment for MSI‐high CRC, with immune checkpoint inhibitors producing durable responses in selected patients. However, this benefit remains largely confined to biologically defined subsets, and most CRCs are microsatellite stable and respond poorly to current immunotherapy approaches [161, 162]. Oncogenic pathways contribute directly to this divergence; for example, Wnt signaling can impair T‐cell priming, whereas TGF‐β promotes immune exclusion through stromal remodeling.

Additional pathways, including MAPK and PI3K signaling, further modulate immune responses by regulating cytokine production and checkpoint expression. These findings indicate that immune resistance is embedded within broader signaling network dysregulation [158]. Combination strategies integrating immunotherapy with targeted therapy, antiangiogenic agents, radiotherapy, or microenvironmental modulation are being investigated to overcome selected components of immune exclusion; however, reliable conversion of MSS CRC into a broadly immunotherapy‐responsive phenotype has not been established.

#### 4.7.4 Mechanisms of Therapeutic Resistance and Pathway Crosstalk

Therapeutic resistance in CRC arises from the plasticity and redundancy of signaling networks. Tumor cells exploit crosstalk and feedback loops to bypass pathway inhibition and maintain survival signaling, while clonal evolution, epithelial–mesenchymal plasticity, stromal barriers, and immune suppression further reinforce resistant phenotypes. Resistance should therefore be understood as a systems‐level process rather than as a single acquired mutation [116, 200].

#### 4.7.5 Emerging Strategies and Future Perspectives

Future CRC therapies are increasingly based on integrative approaches that address signaling complexity and tumor heterogeneity. The central challenge is no longer simply to identify more pathways, but to determine which combinations, sequencing strategies, and biomarkers can convert mechanistic insight into durable clinical benefit [195, 201, 202].

Advances in multiomics, spatial profiling, and real‐time monitoring technologies—including ctDNA and AI‐assisted analytics—are likely to refine this process, but many of these approaches remain investigational and require prospective validation before broad routine adoption [203]. Figure 3 synthesizes the ecological and evolutionary architecture of CRC and highlights the translational therapeutic opportunities that emerge from the interplay among tumor‐intrinsic signaling, microenvironmental regulation, and treatment‐induced selective pressure.

**FIGURE 3:**
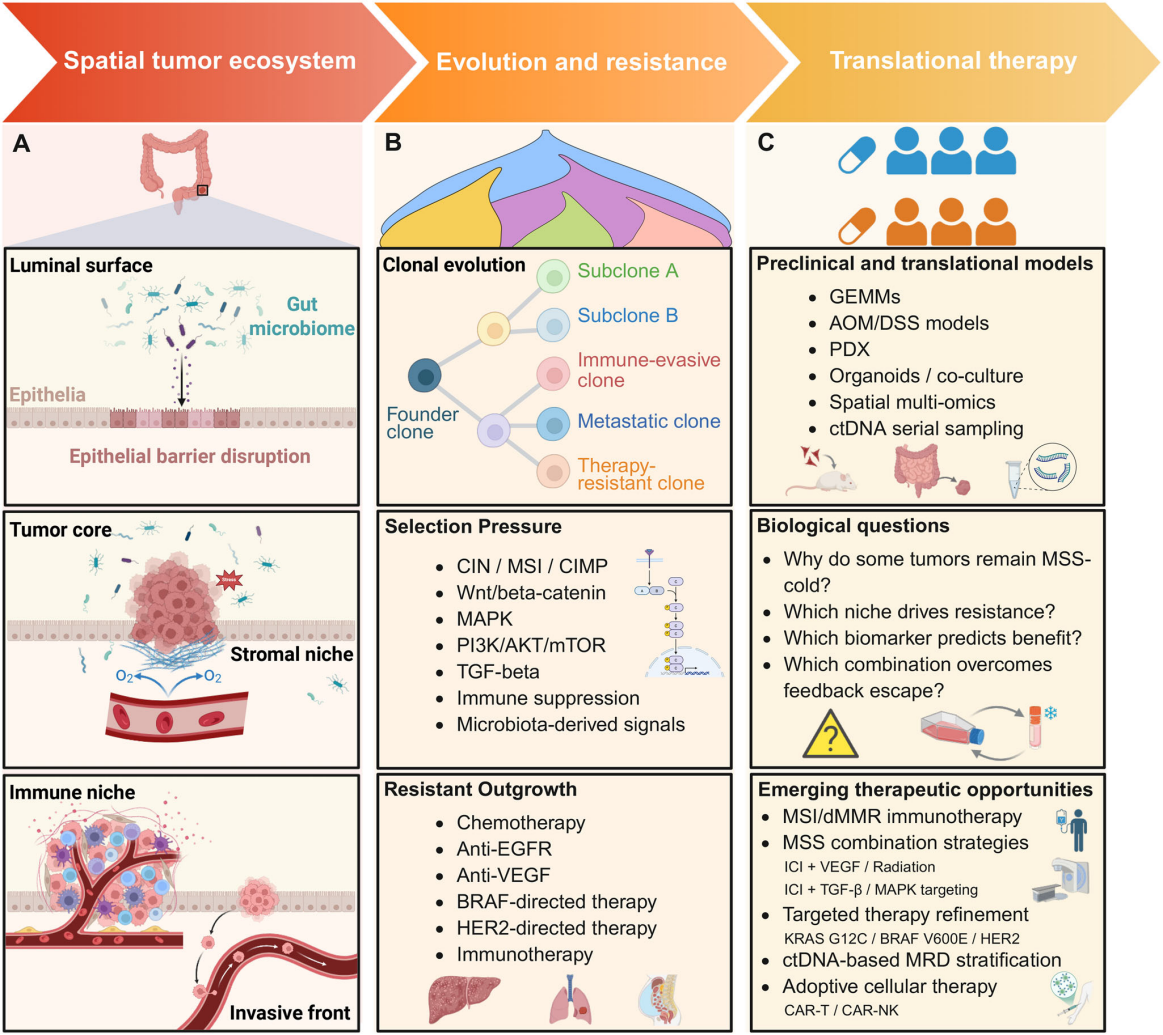
Tumor ecosystem, adaptive evolution, and translational therapeutic opportunities in colorectal cancer. This figure summarizes colorectal cancer (CRC) as a dynamic ecological and evolutionary system. (A) Tumor ecosystem. Colorectal cancer develops within a spatially organized ecosystem composed of tumor cells, immune populations, stromal elements, extracellular matrix, and microbiota‐related signals. Distinct niches across the luminal surface, tumor core, stromal compartments, and invasive front shape immune‐inflamed, immune‐excluded, and mesenchymal‐like states, thereby influencing tumor progression, immune escape, and therapeutic responsiveness. (B) Evolution and resistance. Under genomic instability, pathway crosstalk, and therapeutic pressure, colorectal cancer undergoes continuous clonal diversification and adaptive selection. Interactions among Wnt/β‐catenin, MAPK, PI3K/AKT/mTOR, TGF‐β, immune suppression, and microbiota‐derived signals promote resistant subclones, metastatic dissemination, and treatment failure, highlighting resistance as an ecological and evolutionary process rather than a single molecular event. (C) Translational therapy. Translational platforms including genetically engineered mouse models, inflammation‐associated models, patient‐derived xenografts, organoids, spatial multiomics, and ctDNA‐based monitoring help connect biological mechanisms with clinically actionable strategies. These approaches support biomarker‐guided treatment selection, rational combination therapy, microbiota‐directed intervention, adaptive monitoring of molecular residual disease, and the development of emerging immunotherapeutic and targeted strategies in precision oncology. AKT, protein kinase B; AOM/DSS, azoxymethane/dextran sodium sulfate; BRAF, B‐Raf proto‐oncogene serine/threonine kinase; CAFs, cancer‐associated fibroblasts; CAR‐NK, chimeric antigen receptor natural killer cell; CAR‐T, chimeric antigen receptor T cell; CIMP, CpG island methylator phenotype; CIN, chromosomal instability; CMS, consensus molecular subtype; CRC, colorectal cancer; ctDNA, circulating tumor DNA; dMMR, deficient mismatch repair; ECM, extracellular matrix; EGFR, epidermal growth factor receptor; GEMMs, genetically engineered mouse models; HER2, human epidermal growth factor receptor 2; ICI, immune checkpoint inhibitor; KRAS, Kirsten rat sarcoma viral oncogene homolog; MAPK, mitogen‐activated protein kinase; MRD, molecular residual disease; MSI, microsatellite instability; MSS, microsatellite stable; mTOR, mechanistic target of rapamycin; NK, natural killer; PDX, patient‐derived xenograft; PI3K, phosphoinositide 3‐kinase; TGF‐β, transforming growth factor beta; VEGF, vascular endothelial growth factor; Wnt, Wingless/Int‐1.

## 5 Clinical Features and Disease Presentation

Early‐stage CRC is frequently asymptomatic, which helps explain why symptom‐driven diagnosis often occurs late and why organized screening remains essential. When symptoms do emerge, they are usually nonspecific and can overlap with benign gastrointestinal conditions, contributing to under‐recognition and diagnostic delay [95, 204].

Biological and anatomical factors—including relatively slow early growth, compensatory intestinal function, and the large luminal diameter of the proximal colon—permit tumors to evolve silently for extended periods. As a result, many curable lesions are detected through screening rather than symptom‐triggered evaluation.

### 5.1 Common Presenting Symptoms in Symptomatic Disease

As CRC progresses, patients increasingly present with gastrointestinal and systemic symptoms reflecting tumor growth, mucosal disruption, and luminal compromise. Rectal bleeding is a common manifestation, ranging from occult blood loss to overt hematochezia depending on tumor location and ulceration. Changes in bowel habits—including constipation, diarrhea, or alternating patterns—are frequent and may be accompanied by decreased stool caliber or a sensation of incomplete evacuation [205]. Chronic blood loss can result in iron‐deficiency anemia, particularly in right‐sided tumors, presenting with fatigue, weakness, and pallor. Abdominal pain may arise from inflammation or obstruction, and advanced disease is often associated with weight loss, anorexia, and cachexia. These symptoms are often nonspecific and overlap with benign conditions, contributing to delayed diagnosis and emphasizing the need for clinical vigilance [95].

### 5.2 Clinical Differences by Tumor Location

Clinical presentation varies significantly according to tumor location, reflecting differences in anatomy and tumor biology. Right‐sided colon cancers typically present insidiously, often with occult bleeding and anemia, due to the larger lumen and liquid stool content, leading to delayed diagnosis. In contrast, left‐sided tumors more commonly cause early and overt symptoms, including changes in bowel habits, decreased stool caliber, and visible rectal bleeding, owing to luminal narrowing [206]. Rectal cancers present with characteristic symptoms such as tenesmus, urgency, increased stool frequency, and bright red rectal bleeding, and may cause pelvic pain or urinary symptoms in advanced stages. These clinical differences are accompanied by distinct molecular profiles, with right‐sided tumors more frequently exhibiting MSI‐high status and BRAF mutations, whereas left‐sided tumors are associated with CIN and activation of Wnt/β‐catenin and EGFR signaling pathways [207]. These molecular associations represent population‐level enrichment rather than deterministic categories and should not substitute for direct molecular testing in individual patients.

### 5.3 Distinctive Features in Early‐Onset CRC

EOCRC, defined as diagnosis before 50 years of age, is an increasingly important clinical entity characterized by rising incidence and distinct biological features [90, 208]. Patients are often diagnosed at more advanced stages due to lack of screening and lower clinical suspicion, with symptoms frequently misattributed to benign conditions. This subgroup is associated with more aggressive pathological features and a higher prevalence of hereditary syndromes such as Lynch syndrome, along with distinct molecular and microenvironmental characteristics [209]. Lifestyle and environmental factors, including obesity, dietary patterns, antibiotic exposure, and microbiome‐related features, have been associated with EOCRC risk and are considered plausible contributors; however, their causal roles, critical exposure windows, and relative contributions remain incompletely established. These findings underscore the need for increased awareness and earlier risk‐based screening strategies.

### 5.4 Staging and Patterns of Metastasis

The clinical course and prognosis of CRC are determined by tumor stage and patterns of metastatic dissemination, typically assessed using the TNM classification system. CRC spreads through hematogenous, lymphatic, and transcoelomic routes, with the liver as the most common metastatic site due to portal venous drainage, followed by the lungs, particularly in rectal cancer. Peritoneal dissemination represents a distinct pattern associated with tumor seeding and poor prognosis. Less common sites include bone and brain [7]. Importantly, metastatic patterns are influenced not only by anatomical factors but also by tumor biology and molecular subtype. Understanding these dissemination routes is essential for staging, treatment planning, and surveillance [210].

Taken together, the clinical phenotype of CRC reflects both anatomy and biology. Symptoms often remain absent until tumors are clinically established, yet once present they can still provide important clues regarding tumor location, metastatic pattern, and urgency of evaluation. This combination of silent early evolution and increasingly recognizable patterns of late presentation explains why screening must be tightly linked to structured diagnostic pathways.

## 6 Screening and Early Detection

Building on the heterogeneous and often nonspecific clinical presentation of CRC, particularly the asymptomatic nature of early‐stage disease and the delayed recognition of symptoms, screening has emerged as a cornerstone strategy for reducing CRC incidence and mortality. Reliance on symptom‐driven diagnosis frequently results in detection at more advanced stages, when therapeutic options are limited and prognosis is poorer. In contrast, screening enables identification of both preclinical cancers and premalignant lesions before symptom onset, providing a critical opportunity for early intervention and cancer prevention. The effectiveness of screening is grounded in the well‐characterized adenoma–carcinoma sequence and the prolonged natural history of tumor development, which together create a detectable window for intervention. A range of strategies—including stool‐based tests, endoscopic procedures, and emerging molecular approaches—have been developed and implemented with varying effectiveness across populations. This section reviews the rationale, current practices, and evolving paradigms in CRC screening, with particular emphasis on risk stratification, high‐risk populations, and future directions toward precision screening [211, 212, 213].

### 6.1 Rationale for CRC Screening

Many CRCs arise through a prolonged precursor phase, creating an opportunity for screening and prevention through detection and removal of premalignant lesions. However, natural history varies across conventional adenoma, serrated, inflammation‐associated, hereditary, and less common rapidly progressing pathways. Screening effectiveness therefore depends on both biological detectability and repeated participation over time. This biological basis underlies the development of both stool‐based tests that detect occult bleeding or molecular alterations and endoscopic approaches that enable direct visualization and polypectomy [214].

However, the success of CRC screening depends not only on test performance but also on system‐level factors, including program organization, accessibility, participation, and timely follow‐up. Screening is inherently a multistep process requiring identification of eligible populations, high uptake, prompt diagnostic colonoscopy after positive tests, and quality assurance across centers. Real‐world evidence demonstrates that well‐organized programs can substantially reduce CRC incidence and mortality. In a large integrated US healthcare system, implementation of an organized CRC screening program in which FIT was a major component increased screening participation from 38.9% to 82.7% and was associated with reductions of 25.5% in CRC incidence and 52.4% in CRC mortality [215]. These findings highlight that screening is not merely a diagnostic tool but a comprehensive population‐level preventive strategy [54, 213, 216].

### 6.2 Current Screening Strategies for Average‐Risk Populations

For average‐risk adults, CRC screening strategies are guided by a balance between effectiveness, feasibility, patient acceptability, and healthcare resource allocation [217, 218]. In several US‐based guidelines, average‐risk screening begins at 45 years and generally continues through 75 years, whereas starting ages, stopping ages, test intervals, and preferred modalities vary across jurisdictions, healthcare‐system capacity, and individual risk profiles [219, 220]. In clinical practice, two overarching approaches are commonly adopted: noninvasive, stool‐based screening—typically annual or biennial fecal immunochemical testing (FIT) with colonoscopy reserved for positive results—and primary colonoscopy‐based screening at 10‐year intervals [221]. FIT has become the dominant modality in population‐based programs due to its scalability, cost‐effectiveness, and higher adherence compared with guaiac‐based fecal occult blood testing (gFOBT), while colonoscopy remains the reference standard owing to its ability to directly visualize and remove precancerous lesions, thereby providing both diagnostic and preventive benefits [222].

Additional modalities, including multitarget stool DNA testing (MT‐sDNA), flexible sigmoidoscopy, computed tomography colonography (CTC), and capsule endoscopy, provide complementary options in selected settings [223, 224]. MT‐sDNA improves sensitivity for advanced neoplasia, including nonbleeding lesions, but is limited by lower specificity and higher cost. Flexible sigmoidoscopy reduces incidence and mortality of distal CRC but lacks full colonic coverage. Imaging‐based approaches such as CTC and capsule endoscopy offer minimally invasive alternatives with good patient acceptability, although they require follow‐up colonoscopy for abnormal findings and are constrained by cost, availability, and lack of therapeutic capability. Importantly, no single modality is universally optimal; screening effectiveness depends on achieving high participation rates, adherence to testing intervals, and timely diagnostic follow‐up. These considerations support flexible, context‐specific screening frameworks tailored to healthcare systems and population needs.

#### 6.2.1 FDA‐Approved Blood‐Based Screening as a Participation‐Enhancing Option

Blood‐based CRC screening moved from speculation to US regulated clinical implementation in 2024. In the ECLIPSE study, the cfDNA‐based Shield assay showed 83.1% sensitivity for CRC, 13.2% sensitivity for advanced precancerous lesions, and 89.6% specificity among individuals without advanced neoplasia in an average‐risk screening population undergoing colonoscopy. In July 2024, the FDA approved Guardant Health's Shield blood test for CRC screening in average‐risk individuals aged 45 years or older. The FDA specifies that positive results should be followed by diagnostic colonoscopy and that Shield is not a replacement for diagnostic colonoscopy or for surveillance colonoscopy in high‐risk individuals [14, 17, 23, 225].

Consistent with updated guideline recommendations, blood‐based testing is most appropriately offered to average‐risk individuals who decline or do not complete preferred stool‐based or visual screening tests [24]. This positioning is important because blood‐based testing does not outperform colonoscopy or FIT, but it may improve participation among individuals who remain unscreened with existing modalities. Its role should therefore be viewed as participation‐enhancing rather than as a preventive equivalent to FIT‐ or colonoscopy‐based pathways, particularly given its limited sensitivity for advanced precancerous lesions. Positive blood tests still require diagnostic colonoscopy, and long‐term effects on interval cancer and mortality remain under evaluation [14, 25, 26].

#### 6.2.2 AI‐Assisted Colonoscopy

AI‐assisted colonoscopy is another recent development with potential population‐level implications. Updated randomized evidence and meta‐analytic data indicate that real‐time computer‐aided detection systems can improve adenoma and polyp detection relative to standard colonoscopy.

A 2025 meta‐analysis of 28 randomized trials involving 23,861 participants reported a 20% relative increase in adenoma detection rate and a 55% reduction in adenoma miss rate, although effect sizes varied by system, operator experience, indication mix, and baseline withdrawal quality [27, 28]. The current role of AI is best understood as quality amplification within high‐quality endoscopy programs rather than as a substitute for meticulous colonoscopic technique [29]. Given the expanding range of screening options, Table 2 summarizes the major current and emerging CRC screening modalities, with emphasis on their practical strengths, limitations, and preferred clinical positioning.

**TABLE 2:** Established and recently introduced CRC screening options: Strengths, limitations, and practical positioning.

Modality	Usual interval	Major advantage	Main limitation and best use	References
FIT	Annual or biennial	Scalable, inexpensive, and supported by strong population‐level evidence	Requires repeat adherence; best suited to organized population screening with reliable colonoscopy follow‐up.	[211, 213, 222]
Colonoscopy	About every 10 years	Diagnostic and preventive because visualization and polypectomy occur in one procedure	Invasive and resource intensive; best suited to high‐yield prevention and diagnostic follow‐up.	[11, 221, 222]
Multitarget stool DNA	About every 3 years	Improves noninvasive sensitivity for some advanced lesions	Lower specificity and higher cost; useful when greater noninvasive sensitivity is prioritized.	[24, 223]
CT colonography	About every 5 years	Minimally invasive structural evaluation of the colon	No therapeutic capability and positive findings still need colonoscopy; useful when colonoscopy is not feasible.	[213, 217]
Flexible sigmoidoscopy	About every 5 years	Evidence for distal CRC prevention with lower procedural burden	Incomplete colonic coverage; useful in selected programs or constrained settings.	[11, 217]
Blood‐based cfDNA	Implementation interval and coverage vary by jurisdiction and assay.	Convenient and potentially participation‐enhancing	Low sensitivity for advanced precancerous lesions; positive results require diagnostic colonoscopy; best viewed as an uptake‐enhancing option rather than a preventive equivalent to colonoscopy or FIT.	[14, 17, 23, 24, 25, 26]

Abbreviations: cfDNA, cell‐free DNA; CRC, colorectal cancer; CT, computed tomography; FIT, fecal immunochemical test.

AI‐assisted colonoscopy should be implemented as an adjunct rather than a substitute for meticulous mucosal inspection. Observational evidence has raised concerns that prolonged reliance on computer‐aided detection may be associated with reduced adenoma detection during subsequent unassisted colonoscopy [30]. Because the available evidence is nonrandomized, causality remains uncertain; nevertheless, the finding supports periodic unassisted competency assessment and human‐in‐the‐loop implementation.

### 6.3 Risk‐Adapted and Precision Screening

Traditional CRC screening strategies based primarily on age thresholds fail to capture substantial interindividual variability in risk driven by genetic, environmental, and lifestyle factors. In response, risk‐adapted and precision screening frameworks have been proposed to refine population stratification by integrating determinants such as family history, sex, smoking status, body mass index, dietary patterns, and comorbidities. These approaches aim to allocate screening resources more efficiently by directing higher risk individuals toward colonoscopy and lower risk groups toward less invasive modalities such as FIT [226, 227].

Among available tools, the Asia‐Pacific Colorectal Screening (APCS) score is one of the most widely applied clinical models and performs better than age‐only approaches, although overall discrimination remains moderate. Combining clinical risk factors with stool‐based testing can further improve performance, supporting a hybrid model in which risk stratification guides both modality selection and screening intensity [228].

The field is moving toward multidimensional risk models incorporating polygenic risk scores, circulating biomarkers, microbiota profiles, and digital health data. However, most such approaches remain developmental, and broader clinical adoption will require stronger external validation, implementation studies, and cost‐effectiveness evidence across diverse populations [229].

### 6.4 Screening in High‐Risk Populations

Screening strategies for high‐risk populations differ substantially from those for average‐risk individuals, reflecting increased lifetime risk, earlier disease onset, and distinct biological mechanisms of colorectal carcinogenesis. In these groups, screening is typically initiated at a younger age, performed at shorter intervals, and relies predominantly on colonoscopy due to its high sensitivity and ability to detect and remove premalignant lesions. Individuals with a family history of CRC or advanced adenomas are generally advised to begin screening at 40 years of age or 10 years earlier than the youngest affected relative, with shorter surveillance intervals reflecting elevated risk [230]. In hereditary cancer syndromes, such as Lynch syndrome and FAP, screening is initiated much earlier and conducted more intensively, as these conditions are characterized by accelerated tumorigenesis and markedly increased lifetime risk. For example, Lynch syndrome requires colonoscopic surveillance beginning in early adulthood and repeated every 1–2 years, whereas FAP necessitates endoscopic screening from adolescence, often followed by prophylactic colectomy due to the near certainty of malignant transformation [231, 232, 233].

Patients with IBD involving the colon represent another high‐risk group, in whom chronic inflammation drives carcinogenesis through distinct molecular pathways. In this population, surveillance colonoscopy is typically initiated approximately 8 years after disease onset and individualized according to disease extent, inflammatory burden, and additional risk factors such as primary sclerosing cholangitis or prior dysplasia. Advances in endoscopic techniques, including high‐definition imaging and chromoendoscopy, have improved detection of subtle lesions characteristic of inflammation‐associated neoplasia. Despite the clear benefits of intensified surveillance, adherence remains suboptimal due to limited awareness, access barriers, and healthcare system constraints [234, 235]. Overall, screening in high‐risk populations exemplifies a risk‐stratified and biologically informed approach to cancer prevention, aligning screening intensity with underlying disease risk to enable earlier detection and improved clinical outcomes.

### 6.5 Global Screening Programs and Coverage Inequalities

The implementation of CRC screening programs varies markedly across regions, reflecting disparities in healthcare infrastructure, economic resources, policy prioritization, and population awareness [50, 236, 237]. Although an increasing number of countries—particularly in Europe, North America, and parts of East Asia—have established organized, population‐based screening programs, global coverage remains highly uneven [218]. A recent synthesis identified national CRC screening programs in 45 countries, with coverage data available for 33; reported participation ranged from approximately 1% in some settings to nearly 80% in established Northern European programs [50]. Stool‐based testing, primarily FIT, remains the most widely adopted first‐line modality due to its scalability and cost‐effectiveness. However, screening participation is influenced by demographic and socioeconomic factors, generally increasing with age and being slightly higher among women. Importantly, the effectiveness of screening depends not only on test performance but also on program coverage, adherence, and continuity of care [54]. In resource‐limited settings, barriers such as inadequate infrastructure, workforce shortages, competing health priorities, and low awareness significantly constrain implementation, while socioeconomic inequalities, cultural perceptions, and logistical challenges further contribute to uneven uptake. Addressing these disparities requires context‐specific strategies, including integration of screening into primary care, use of cost‐effective modalities, public education, and sustained policy commitment, highlighting the importance of improving global equity in CRC prevention.

### 6.6 Challenges and Barriers to Screening Uptake

Despite strong evidence supporting the effectiveness of CRC screening, participation remains suboptimal in many settings, limiting its impact on population health [54, 238, 239]. A key challenge is the asymptomatic nature of early disease, which reduces perceived urgency and engagement among eligible individuals. Barriers to screening operate at multiple levels, including individual factors such as limited awareness, fear of invasive procedures, and cultural perceptions; provider‐related factors such as inconsistent recommendations and communication constraints; and system‐level issues including economic limitations, geographic disparities, and lack of standardized screening pathways [240]. In addition, organized screening programs require substantial infrastructure and coordination, while opportunistic screening may fail to reach underserved populations, highlighting trade‐offs between these approaches [241]. Screening uptake is also strongly influenced by socioeconomic status, education, and ethnicity, with lower participation observed in disadvantaged groups. Addressing these challenges requires integrated and context‐specific strategies, including public education, patient navigation systems, use of less invasive modalities, and digital health interventions to improve adherence and follow‐up [242]. Ultimately, overcoming these barriers is essential for translating the proven efficacy of CRC screening into real‐world reductions in incidence and mortality and for reducing global disparities in cancer outcomes.

### 6.7 Future Directions in CRC Screening

The future of CRC screening is shifting from uniform, age‐based approaches toward multimodal, risk‐adapted frameworks that align screening strategies with individual risk profiles and healthcare contexts [211, 243]. Advances in clinical risk prediction models and integration of demographic, lifestyle, and familial factors enable more precise stratification of populations, allowing low‐risk individuals to undergo noninvasive testing such as FIT, while higher risk individuals are prioritized for colonoscopy [244]. This stratified approach improves both detection efficiency and resource allocation, supporting more personalized and sustainable screening programs.

In parallel, emerging technologies—including blood‐based assays, multitarget molecular testing, and AI‐assisted tools—are expected to enhance early detection and screening adherence, although most remain under evaluation for routine use [25, 245]. Future progress will depend on integrating these innovations into scalable and cost‐effective healthcare systems while maintaining equitable access across populations [23, 31, 242]. Overall, CRC screening is evolving toward a more personalized, minimally invasive, and system‐integrated paradigm, forming a critical bridge between early detection and subsequent diagnostic and therapeutic pathways. Figure 4 outlines the integrated clinical continuum spanning risk stratification and screening, diagnostic and molecular workup, stage‐ and biomarker‐informed therapeutic decision‐making, and longitudinal surveillance.

**FIGURE 4:**
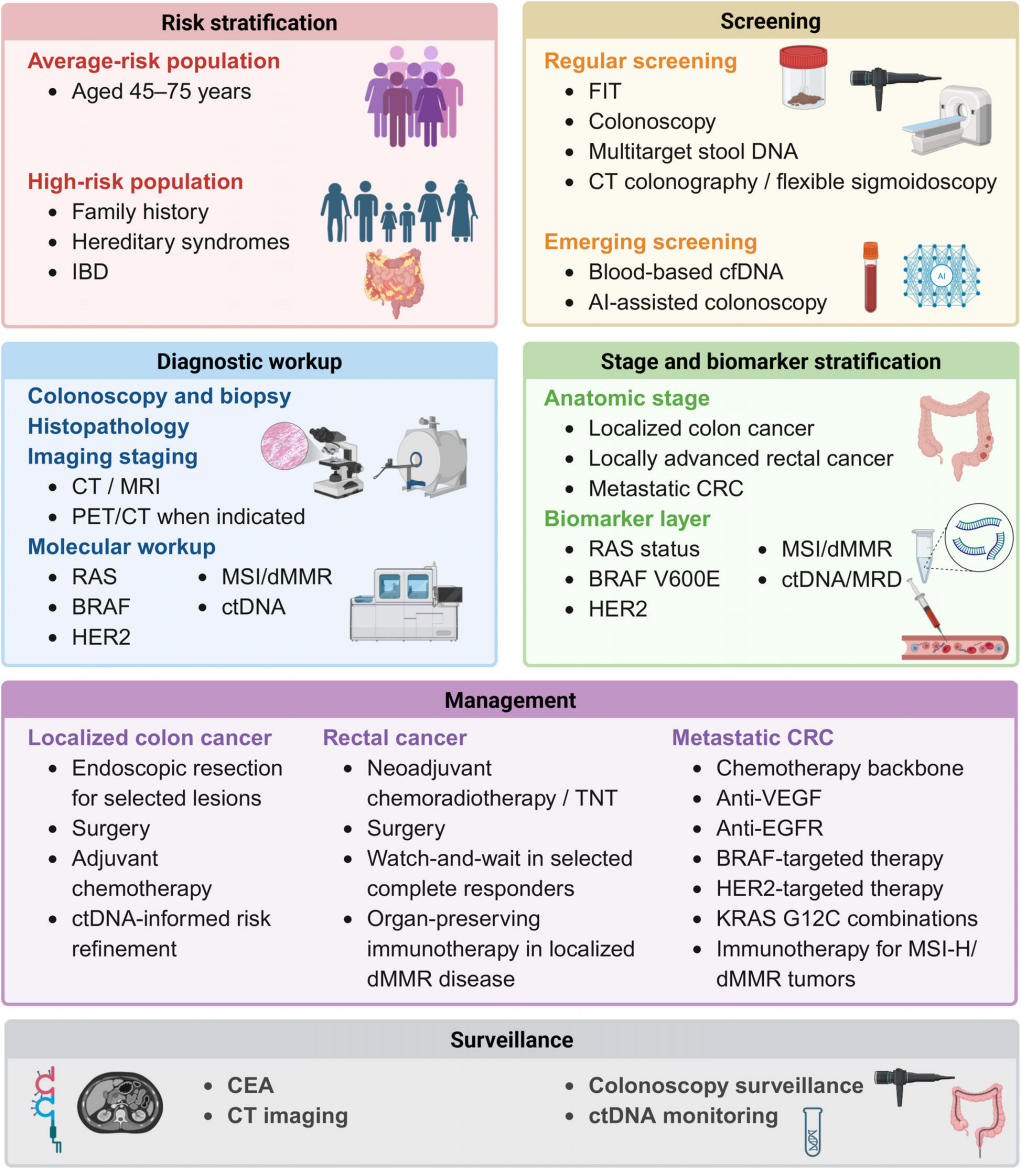
Integrated clinical pathway for colorectal cancer screening, diagnosis, and stage‐ and biomarker‐guided management. This figure summarizes the clinical continuum of colorectal cancer (CRC) care, from risk stratification and screening to diagnostic workup, molecular characterization, treatment selection, and longitudinal surveillance. Screening includes stool‐based tests, colonoscopy, blood‐based assays, and AI‐assisted endoscopy. Diagnostic evaluation integrates colonoscopy, histopathology, imaging, and molecular profiling, including RAS, BRAF, HER2, MSI/dMMR, and ctDNA assessment. Clinical management is guided by both disease stage and biomarker status, encompassing endoscopic or surgical treatment for localized disease, multimodal strategies for rectal cancer, and chemotherapy, targeted therapy, or immunotherapy for metastatic CRC. Longitudinal surveillance integrates imaging, colonoscopy, and serum markers, while ctDNA may provide additional recurrence‐risk information and support clinical‐trial enrollment. Treatment initiated solely on the basis of molecular recurrence remains investigational. AI, artificial intelligence; BRAF, B‐Raf proto‐oncogene serine/threonine kinase; CEA, carcinoembryonic antigen; cfDNA, cell‐free DNA; CRC, colorectal cancer; CT, computed tomography; ctDNA, circulating tumor DNA; dMMR, deficient mismatch repair; EGFR, epidermal growth factor receptor; FIT, fecal immunochemical test; HER2, human epidermal growth factor receptor 2; IBD, inflammatory bowel disease; KRAS, Kirsten rat sarcoma viral oncogene homolog; MRD, molecular residual disease; MRI, magnetic resonance imaging; MSI, microsatellite instability; PET/CT, positron emission tomography/computed tomography; RAS, rat sarcoma viral oncogene homolog family; TNT, total neoadjuvant therapy; VEGF, vascular endothelial growth factor.

## 7 Diagnosis and Molecular Workup

Following screening detection or the onset of clinical symptoms, diagnosis and molecular characterization of CRC represent critical steps in confirming disease, defining stage, and guiding individualized treatment [246]. Contemporary diagnostic evaluation increasingly integrates morphology, histopathology, imaging, and molecular information rather than relying on anatomy alone [8, 247].

Endoscopy enables direct visualization and tissue acquisition, while histopathology provides definitive confirmation of malignancy and key prognostic features, including tumor differentiation and invasion patterns. Imaging modalities such as CT and MRI define local extent and metastatic spread, forming the basis of TNM staging and treatment planning. Beyond these foundational tools, molecular profiling has become central to clinical practice. Universal tumor MMR/MSI assessment should be distinguished from treatment‐oriented molecular profiling in advanced or metastatic disease. Extended RAS and BRAF V600E testing is central to treatment selection in mCRC, whereas HER2 and selected rare‐fusion testing are performed in appropriate advanced‐disease contexts [248, 249].

Emerging technologies are further reshaping the diagnostic landscape. Liquid biopsy enables minimally invasive detection of circulating tumor components, supporting real‐time monitoring of tumor dynamics and minimal residual disease. In parallel, artificial intelligence (AI) and radiomics are increasingly applied to integrate imaging, clinical, and molecular data, enhancing diagnostic accuracy and predictive modeling [250, 251].

Collectively, these advances reflect a shift from a purely anatomical model to an integrated, systems‐level diagnostic paradigm [246, 252]. This approach improves staging accuracy and provides the foundation for personalized treatment strategies [253, 254]. The following sections outline the diagnostic pathway, key modalities, and emerging tools that define modern CRC diagnosis.

In practical terms, the contemporary diagnostic pathway should be read as a layered process. Histopathological confirmation and anatomical staging remain indispensable foundations, and for many patients they still determine the first major management decisions. Molecular workup then adds a second layer that can redirect therapy selection, identify hereditary implications, or clarify prognosis. Emerging tools such as ctDNA, radiomics, and AI‐based prediction should be viewed as potential complements to this pathway rather than replacements for established diagnostic steps [250, 255]. Their greatest near‐term value may lie in refining risk estimates, anticipating resistance, and supporting longitudinal monitoring in carefully defined clinical contexts.

### 7.1 Diagnostic Pathway After Positive Screening or Symptoms

The diagnostic pathway for CRC is typically initiated after a positive screening result—such as FIT, stool DNA testing, or other noninvasive modalities—or the emergence of alarm symptoms, including rectal bleeding, iron‐deficiency anemia, unexplained weight loss, or persistent changes in bowel habits [256, 257]. In screening‐detected cases, a positive noninvasive test requires prompt colonoscopy, which serves as the definitive diagnostic step by enabling lesion visualization, biopsy, and potential therapeutic intervention [241]. Timely diagnostic colonoscopy after a positive stool‐based test is critical, because longer delays—particularly delays approaching or exceeding approximately 9–12 months in observational studies—have been associated with a higher likelihood of CRC and more advanced‐stage disease. In symptomatic patients, colonoscopy remains the primary diagnostic modality but may be preceded by initial clinical evaluation, including laboratory testing and, in selected cases, imaging to assess complications such as obstruction or metastatic disease [258].

The diagnostic process therefore follows a structured progression from clinical suspicion to confirmation, integrating symptom assessment, risk evaluation, and appropriate use of diagnostic resources. Effective coordination across primary care, gastroenterology, radiology, and pathology is essential for timely and accurate diagnosis. System‐level factors also play a crucial role. Efficient referral pathways, standardized algorithms, and access to diagnostic services are necessary to minimize delays [259]. Disparities in access—driven by socioeconomic and geographic factors—can significantly affect diagnostic timeliness and outcomes. Overall, the diagnostic pathway represents a critical bridge between early detection and definitive management, with direct impact on staging accuracy, treatment decisions, and patient prognosis.

### 7.2 Endoscopy and Histopathological Confirmation

Colonoscopy remains the gold standard for CRC diagnosis, offering direct mucosal visualization, targeted tissue acquisition, and, in selected cases, immediate therapeutic intervention [260]. High‐definition endoscopy enables detailed evaluation of lesion characteristics, including size, morphology (polypoid, flat, or depressed), surface pattern, and extent of luminal involvement, which are critical for diagnostic assessment and treatment planning. Advanced imaging techniques—such as narrow‐band imaging (NBI), chromoendoscopy, and magnification endoscopy—further improve detection and characterization of subtle or flat lesions, particularly sessile serrated lesions that may be missed with conventional techniques [261]. Definitive diagnosis relies on histopathological confirmation obtained through biopsy or resection specimens. Pathological evaluation confirms malignancy and provides key prognostic information, including tumor differentiation, depth of invasion, lymphovascular and perineural invasion, tumor budding, and resection margin status [262]. These features are essential for staging, risk stratification, and therapeutic decision‐making.

In early‐stage disease, histological assessment also determines the adequacy of endoscopic resection and the need for additional surgery. Moreover, pathological specimens serve as the basis for molecular and immunohistochemical analyses, including assessment of MMR status and mutations in RAS, BRAF, and other actionable genes, thereby linking diagnosis directly to precision therapy. The diagnostic accuracy of colonoscopy depends heavily on procedural quality [263]. Adequate bowel preparation, complete examination of the colon, and adherence to quality indicators—such as adenoma detection rate and withdrawal time—are critical to minimizing missed lesions. Inadequate preparation or incomplete visualization, particularly in the proximal colon, contributes to interval cancers. Therefore, standardized quality control and continuous improvement in endoscopic practice are essential components of effective CRC diagnosis.

Collectively, integration of high‐quality endoscopy with detailed histopathological and molecular evaluation forms the cornerstone of modern CRC diagnosis, bridging morphological assessment with precision oncology [247].

### 7.3 Imaging Evaluation and Clinical Staging

Imaging plays a central role in the staging and management of CRC by defining tumor extent, resectability, and metastatic spread [246, 264, 265]. Within the TNM framework, imaging is used to assess primary tumor invasion (T), regional lymph node involvement (N), and distant metastases (M), thereby guiding prognostic stratification and treatment planning. In clinical practice, a multimodal approach is adopted. Cross‐sectional imaging provides comprehensive evaluation of systemic disease, while specialized techniques offer high‐resolution assessment of local structures, particularly in rectal cancer. Beyond staging, imaging findings directly influence treatment decisions, including selection of surgery, neoadjuvant therapy, and metastasis‐directed interventions. Imaging is also essential for radiotherapy planning, response assessment, and post‐treatment surveillance. Recent advances—including high‐resolution MRI, functional imaging, and hybrid modalities—have improved diagnostic accuracy and enabled noninvasive characterization of tumor biology [266]. Emerging approaches such as radiomics and AI further enhance image‐based risk prediction and treatment stratification.

Collectively, imaging serves as a cornerstone of CRC management, linking anatomical assessment with clinical decision‐making and enabling a more precise and individualized approach to care [250].

#### 7.3.1 CT

Contrast‐enhanced CT of the chest, abdomen, and pelvis is the first‐line imaging modality for CRC staging, particularly for evaluation of distant metastatic disease. Its wide availability, rapid acquisition, and high spatial resolution allow comprehensive assessment of overall tumor burden [246, 267]. CT is especially effective for detecting liver metastases, the most common site of CRC spread, and for identifying pulmonary lesions, lymphadenopathy, and extrahepatic disease. It is also essential in evaluating complications such as obstruction, perforation, and abscess formation. In addition to initial staging, CT is widely used for treatment planning and longitudinal monitoring, including response assessment using standardized criteria such as RECIST and surveillance after curative treatment. However, CT has limitations in evaluating early T‐stage disease and local invasion, particularly in rectal cancer, where detailed assessment of the mesorectal fascia and circumferential resection margin is required. It is also less sensitive for small peritoneal metastases [268]. Other limitations include radiation exposure and the need for iodinated contrast. Emerging techniques, such as dual‐energy CT and AI‐assisted analysis, may improve lesion detection and provide functional insights [250, 269]. Overall, CT remains the foundational imaging modality in CRC staging, complemented by other techniques for detailed local or functional assessment.

#### 7.3.2 MRI

MRI is the preferred modality for local staging of rectal cancer due to its superior soft‐tissue contrast and ability to delineate critical anatomical structures [265, 270]. High‐resolution pelvic MRI enables precise assessment of tumor depth, mesorectal fascia involvement, circumferential resection margin, and extramural vascular invasion. These features are key determinants of surgical planning and the use of neoadjuvant therapy, making MRI the gold standard for rectal cancer staging. MRI also plays a complementary role in evaluating hepatic metastases, particularly when CT findings are inconclusive [266]. Techniques such as diffusion‐weighted imaging and hepatocyte‐specific contrast agents improve detection of small lesions and assessment of resectability. Limitations include longer acquisition time, higher cost, and limited availability. Image quality may also be affected by motion or patient tolerance [271]. Despite these constraints, advances in functional MRI and quantitative imaging are expanding its role beyond anatomical assessment to include evaluation of tumor biology and treatment response.

#### 7.3.3 PET/CT When Indicated

PET/CT provides functional imaging based on tumor metabolic activity and is used selectively in CRC. It is not recommended for routine initial staging but is valuable in specific clinical scenarios [272, 273]. Its main applications include evaluation of indeterminate lesions on CT or MRI, detection of occult metastases, and assessment of suspected recurrence, particularly in patients with rising CEA levels and negative conventional imaging [274, 275]. PET/CT is particularly useful for identifying extrahepatic disease that may alter surgical planning and prevent unnecessary interventions. It may also assist in treatment response assessment in advanced disease. However, limitations include reduced sensitivity for small lesions and mucinous tumors, as well as false positives due to inflammation. Higher cost, limited availability, and radiation exposure further restrict routine use. Therefore, PET/CT is best considered an adjunctive modality within a multimodal imaging strategy, providing additional metabolic information when it is likely to influence management decisions.

### 7.4 Biomarkers and Molecular Profiling

Molecular profiling has become a central component of CRC management, shifting diagnosis from a purely anatomical framework to a biologically informed precision oncology paradigm [247]. Advances in genomic technologies have enabled identification of key driver alterations that inform both tumor biology and clinical decision‐making [8, 252]. In current practice, routine testing includes RAS (KRAS/NRAS), BRAF, HER2, and MMR/MSI status, each with direct therapeutic implications. RAS mutations predict resistance to anti‐EGFR therapy, BRAF mutations define an aggressive subtype requiring combination strategies, HER2 amplification identifies candidates for HER2‐targeted therapy, and MSI/dMMR status predicts responsiveness to immune checkpoint inhibitors [198].

Beyond these core biomarkers, comprehensive profiling using NGS increasingly captures rare but actionable alterations and broader genomic signatures, linking molecular features with tumor heterogeneity, evolution, and CMS classification [99]. However, challenges remain, including cost, accessibility, standardization, and interpretation of complex genomic data, as well as spatial and temporal heterogeneity between primary and metastatic lesions. Despite these limitations, molecular profiling now underpins precision treatment strategies and continues to expand in scope [276].

#### 7.4.1 RAS

RAS mutations, primarily involving KRAS and NRAS, occur in approximately 40%–50% of CRC and represent key predictive biomarkers in advanced disease. These mutations result in constitutive activation of the EGFR/RAS/RAF/MEK/ERK pathway, rendering tumors resistant to anti‐EGFR therapies such as cetuximab and panitumumab [247, 277]. Consequently, RAS testing is mandatory prior to initiation of anti‐EGFR treatment, with therapy restricted to RAS wild‐type tumors. Beyond treatment selection, RAS mutations are also associated with adverse clinical features and reduced sensitivity to anti‐EGFR therapy in relevant settings. Importantly, RAS status is dynamic, as acquired resistance may emerge through secondary mutations under therapeutic pressure, highlighting the value of serial monitoring with ctDNA [278, 279]. Comprehensive testing across multiple exons using NGS further improves detection accuracy and molecular stratification.

#### 7.4.2 BRAF

BRAF mutations, most commonly V600E, occur in approximately 5%–10% of CRC and define a biologically distinct subgroup with aggressive behavior and poor prognosis [280]. These mutations drive constitutive MAPK pathway activation and are associated with right‐sided tumors, high‐grade histology, and increased peritoneal metastasis [8]. Although BRAF V600E mutation does not by itself define the same resistance category as RAS mutation, responses to anti‐EGFR monotherapy are limited, supporting combination regimens targeting BRAF and EGFR, with or without MEK inhibition. BRAF status also contributes to molecular classification, often aligning with CMS1, and plays a role in differentiating sporadic MSI‐high tumors from Lynch syndrome–associated disease.

#### 7.4.3 HER2

HER2 amplification or overexpression, present in approximately 2%–5% of CRC, represents an actionable target, particularly in RAS/BRAF wild‐type tumors. HER2 activation promotes downstream MAPK and PI3K/AKT signaling and is associated with resistance to anti‐EGFR therapy [198, 281]. Clinically, HER2‐positive CRC may benefit from HER2‐targeted approaches, including monoclonal antibodies, antibody–drug conjugates, and dual blockade strategies [282]. Accurate detection relies on IHC and ISH‐based methods, although testing criteria continue to evolve in CRC‐specific contexts. Despite its relatively low prevalence, HER2 exemplifies the expanding role of targeted therapy in molecularly defined subgroups.

#### 7.4.4 MSI/dMMR

MSI and dMMR represent critical biomarkers with diagnostic, prognostic, and therapeutic significance [247, 283, 284]. Loss of MMR function leads to accumulation of mutations and high tumor mutational burden, resulting in increased neoantigen formation and a highly immunogenic TME. Clinically, MSI‐high tumors are associated with favorable prognosis in early‐stage disease and demonstrate robust responses to immune checkpoint inhibitors, making MSI/dMMR a key predictive biomarker for immunotherapy [285]. In addition, MMR/MSI testing serves as an initial tumor‐based screen for possible Lynch syndrome. Definitive evaluation may require MLH1 promoter methylation analysis, BRAF V600E testing in appropriate MLH1‐deficient tumors, genetic counseling, and germline testing. Detection is routinely performed using IHC or molecular assays and is recommended in all newly diagnosed CRC cases. However, as most CRCs are MSS, expanding immunotherapy benefit remains a major challenge.

#### 7.4.5 NTRK Fusions and Other Rare Potentially Actionable Alterations

Rare genomic alterations, including NTRK fusions and other targetable events such as ALK, ROS1, and RET rearrangements, represent emerging therapeutic opportunities in CRC, particularly in advanced or refractory disease. Although their prevalence is low, these alterations are clinically significant due to high sensitivity to targeted inhibitors, such as TRK inhibitors, which demonstrate tumor‐agnostic efficacy [286, 287, 288]. Detection requires comprehensive genomic profiling, typically via NGS, capable of identifying diverse mutation types and gene fusions. The identification of these rare alterations reflects a broader shift toward tumor‐agnostic treatment strategies guided by molecular features rather than tumor origin [110]. However, challenges remain in implementation, including cost, accessibility, and limited CRC‐specific evidence.

### 7.5 Liquid Biopsy and Emerging Biomarkers

Liquid biopsy technologies are reshaping the diagnostic and monitoring landscape of CRC by enabling minimally invasive detection of tumor‐derived components in blood and other body fluids [248, 289]. Unlike tissue biopsy, which provides a static and site‐specific snapshot, liquid biopsy offers a dynamic view of tumor burden, heterogeneity, and evolution.

Clinically, liquid biopsy has potential applications in early detection, MRD assessment, treatment monitoring, and identification of resistance mechanisms [253]. Among currently available analytes, ctDNA is the closest to routine clinical integration in selected settings, whereas circulating tumor cells, extracellular vesicles (EVs), and methylation‐based assays remain more exploratory and method‐dependent.

#### 7.5.1 ctDNA

ctDNA, a fraction of cell‐free DNA released from tumor cells, is one of the most clinically relevant liquid biopsy biomarkers in CRC [15]. It carries tumor‐specific alterations, including mutations, copy number changes, and methylation patterns, and serves as a dynamic indicator of tumor burden. ctDNA has demonstrated strong prognostic value in MRD detection, recurrence prediction, and treatment monitoring, with postoperative ctDNA positivity strongly associated with increased recurrence risk and often preceding radiological detection. In addition, ctDNA enables real‐time tracking of tumor evolution, including emergence of resistance mutations such as acquired RAS alterations during anti‐EGFR therapy, thereby supporting molecular monitoring, resistance assessment, and research on biomarker‐informed treatment sequencing or anti‐EGFR rechallenge in selected settings [279]. Despite these advantages, sensitivity remains limited in early‐stage disease and assay standardization is lacking, but ongoing improvements in sequencing technologies and multianalyte approaches are expected to enhance clinical applicability.

The strongest recent advance in liquid biopsy has been the transition of ctDNA from a predominantly prognostic research tool to a candidate decision‐support biomarker for molecular residual disease. The GALAXY arm of CIRCULATE‐Japan, reported in 2024, demonstrated in 2240 resectable CRC patients that postoperative ctDNA positivity identified substantially higher recurrence risk and worse survival, while ctDNA clearance after adjuvant therapy was associated with improved outcomes. These observational findings reinforce the clinical validity of postoperative ctDNA as a prognostic marker and suggest that longitudinal ctDNA dynamics may provide additional risk information. However, associations between ctDNA clearance, adjuvant chemotherapy, and improved outcomes should not be interpreted as proof that ctDNA‐directed treatment improves survival [32].

#### 7.5.2 CTCs

CTCs are intact tumor cells circulating in the bloodstream and provide insight into metastatic dissemination and tumor biology [248, 290]. Unlike ctDNA, CTCs retain cellular structure, enabling analysis of morphology, protein expression, and functional characteristics. CTC counts correlate with disease burden and prognosis, with higher levels associated with poorer outcomes in metastatic CRC. Advances in detection technologies—including EpCAM‐based capture, size‐based methods, and microfluidic platforms—have improved sensitivity, while molecular characterization allows assessment of EMT status and resistance mechanisms [290]. However, clinical application remains limited by low abundance in early disease, technical variability, and lack of standardized thresholds. Integration with other liquid biopsy components may enhance their clinical utility [248].

#### 7.5.3 Methylation‐Based Assays

DNA‐methylation assays represent an important class of epigenetic biomarkers, exploiting early tumor‐associated methylation changes for CRC detection [291, 292, 293]. Among these, SEPT9 methylation testing is one of the most extensively evaluated blood‐based epigenetic assays for CRC detection, but its role in organized screening remains limited by variable sensitivity, specificity, and precursor‐lesion detection [294]. These approaches offer advantages in detecting cancer independent of bleeding and may improve participation in noninvasive screening. However, sensitivity and specificity remain variable, particularly for early‐stage disease and advanced precursor lesions, limiting their use as stand‐alone tools. Ongoing development of multimarker panels combining genetic and epigenetic signals is expected to improve performance and broaden applicability [295, 296].

#### 7.5.4 Extracellular Vesicles

EVs, including exosomes and microvesicles, are lipid‐bound particles released by tumor cells that carry nucleic acids, proteins, and metabolites reflective of tumor biology [297, 298]. EVs are highly stable in circulation and play active roles in tumor progression, including immune modulation, angiogenesis, and metastatic niche formation [299]. From a diagnostic perspective, EV‐associated molecular signatures—such as microRNAs and protein profiles—have shown potential for early detection, monitoring, and prognostic assessment [300, 301]. However, clinical translation is limited by challenges in standardization, isolation methods, and assay reproducibility. Advances in nanotechnology and high‐throughput analysis are expected to improve feasibility and enable integration into broader liquid biopsy strategies.

#### 7.5.5 Randomized Evidence and Clinical Caveats for ctDNA‐Informed Management

Despite its strong prognostic performance, ctDNA should not be interpreted as a universally decisive treatment biomarker [255, 289]. False‐negative results may occur in tumors with low ctDNA shedding, after successful local therapy, or when blood sampling is performed at suboptimal time points. Conversely, ctDNA positivity may precede radiological recurrence by several months, but early molecular detection does not by itself identify an effective therapeutic intervention. Assay design, sampling time, tumor stage, recent surgery or chemotherapy, and biological differences in ctDNA shedding must therefore be considered when interpreting results.

Randomized evidence indicates that the clinical utility of ctDNA is stage‐ and strategy‐specific. In the DYNAMIC trial of Stage II colon cancer, a ctDNA‐guided approach reduced adjuvant chemotherapy use while maintaining similar 5‐year recurrence‐free survival (88% vs. 87%) and overall survival (93.8% vs. 93.3%) compared with standard management [33]. By contrast, in the DYNAMIC‐III trial of Stage III colon cancer, ctDNA‐guided de‐escalation in ctDNA‐negative patients reduced oxaliplatin exposure but did not meet the prespecified noninferiority criterion for 3‐year recurrence‐free survival (85.3% vs. 88.1%). Treatment escalation in ctDNA‐positive patients also did not improve 2‐year recurrence‐free survival compared with standard management (51% vs. 61%) [34]. The ALTAIR Phase 3 trial further evaluated treatment initiated at molecular recurrence. Among 243 patients who were ctDNA positive after curative‐intent treatment but had no radiological evidence of disease, trifluridine/tipiracil did not significantly improve the primary disease‐free survival endpoint compared with placebo (median, 9.30 vs. 5.55 months; hazard ratio, 0.79; 95% confidence interval, 0.60–1.05; *p* = 0.107) [35]. Grade 3 or higher hematological adverse events occurred in 73.0% and 3.3% of patients, respectively. These findings indicate that treatment initiated solely because of ctDNA‐defined molecular recurrence cannot currently be regarded as an established surveillance strategy. Postoperative ctDNA should therefore be used for prognostic stratification, selected stage‐specific decision support, and clinical‐trial enrollment. It should not routinely replace clinicopathological criteria or independently trigger treatment escalation, de‐escalation, omission, or intervention at molecular recurrence.

### 7.6 Radiomics and AI‐Assisted Diagnosis

Radiomics and AI are emerging as investigational decision‐support tools in CRC diagnosis, enabling extraction of high‐dimensional quantitative features from medical imaging beyond conventional visual assessment [250, 302]. Radiomics converts standard imaging data—such as CT, MRI, and PET—into analyzable datasets that capture tumor shape, texture, intensity, and spatial heterogeneity, providing noninvasive insights into tumor biology. These features have shown potential in improving tumor characterization, staging accuracy, risk stratification, and prediction of treatment response, particularly in rectal cancer [303]. In parallel, AI‐based approaches, including machine learning and deep learning, facilitate integration of imaging with clinical, pathological, and molecular data, generating predictive models for diagnosis, prognosis, and treatment planning. Emerging applications include AI‐assisted polyp detection during colonoscopy, automated tumor segmentation, and prediction of molecular subtypes or biomarker status directly from imaging [31, 304].

Despite these advances, clinical implementation remains limited [250, 305]. Radiomic features are sensitive to variability in imaging protocols, scanner types, and data preprocessing, leading to challenges in reproducibility and generalizability. The lack of standardized pipelines for acquisition, feature extraction, and model validation further hinders clinical translation [306]. In addition, many studies remain retrospective and single‐center, with limited external validation. Ethical considerations, data privacy, and model interpretability also require careful attention. Future progress will depend on standardized frameworks, large‐scale multicenter validation, and integration into clinical workflows [307]. The integration of radiomics, multiomics, and digital health data may support multimodal clinical decision‐making, but routine use requires prospective demonstration of calibration, external validity, incremental clinical utility, workflow benefit, and robustness to data drift and algorithmic bias [254]. To synthesize the biomarkers most relevant to current clinical decision‐making, Table 3 summarizes the core molecular markers in contemporary CRC management, their principal clinical implications, and their recent translational relevance.

**TABLE 3:** Core biomarkers in contemporary CRC management and their current clinical implications.

Biomarker	Approximate role in CRC	Current clinical implication	Recent translational relevance	References
RAS (KRAS/NRAS)	Common MAPK‐activating alterations	Predict lack of benefit from anti‐EGFR therapy in mCRC	ctDNA increasingly supports monitoring of emergent RAS‐mediated resistance, while EGFR‐combined KRAS G12C inhibitor regimens have become FDA‐approved later‐line targeted options in the United States for previously treated KRAS G12C‐mutated advanced or metastatic CRC.	[43, 44, 247, 277, 278, 279, 308, 309]
BRAF V600E	Aggressive molecular subtype	Guides BRAF/EGFR‐based therapy and prognostic stratification	BRAF/EGFR blockade combined with fluorouracil‐based chemotherapy has shown first‐line clinical benefit and is FDA‐approved in the United States for adult patients with BRAF V600E‐mutant mCRC detected by an FDA‐authorized test.	[39, 280, 310, 311]
HER2 amplification or overexpression	Actionable anti‐EGFR‐resistant subset	Supports HER2‐directed therapy in selected refractory mCRC	Antibody–drug conjugates broaden the therapeutic relevance of HER2 testing.	[41, 42, 281, 282]
MSI‐H/dMMR	Hypermutated and immune‐responsive subtype	Predicts strong likelihood of benefit from immune checkpoint blockade	Localized dMMR disease now intersects with organ‐preserving immunotherapy strategies.	[36, 37, 38, 283, 284, 285, 312, 313]
ctDNA/MRD	Postoperative prognostic and dynamic molecular biomarker	Refines recurrence‐risk stratification after curative‐intent treatment; treatment‐guiding utility remains stage‐ and strategy‐dependent	DYNAMIC supports reduced chemotherapy use in selected Stage II colon cancer, whereas DYNAMIC‐III and ALTAIR did not validate routine Stage III escalation/de‐escalation or treatment initiated solely at molecular recurrence.	[15, 32, 33, 34, 35, 249, 314, 315]
Rare fusions and low‐frequency alterations	Low‐prevalence actionable targets	May enable tumor‐agnostic therapy in advanced disease	Support the rationale for broader NGS panels in selected advanced or refractory settings.	[286, 287, 288, 316, 317]

Abbreviations: ctDNA, circulating tumor DNA; dMMR, deficient mismatch repair; EGFR, epidermal growth factor receptor; HER2, human epidermal growth factor receptor 2; MAPK, mitogen‐activated protein kinase; mCRC, metastatic colorectal cancer; MRD, molecular residual disease; MSI‐H, microsatellite instability‐high; NGS, next‐generation sequencing.

Overall, modern CRC diagnosis is no longer defined solely by histological confirmation and anatomic staging [8, 246, 247]. The clinically decisive task is to integrate endoscopy, pathology, imaging, and molecular information quickly enough to influence treatment sequencing, surveillance intensity, and access to biomarker‐directed therapies [248, 252]. That integrated diagnostic logic provides the foundation for the stage‐specific and biologically stratified management strategies discussed in the next section.

## 8 Management of Colorectal Cancer

The management of CRC is guided by a stage‐based and biologically stratified framework that integrates anatomical staging with molecular profiling and patient‐specific factors [247, 318]. Traditionally, treatment relied on surgical resection for localized disease and cytotoxic chemotherapy for advanced stages. However, advances in surgical techniques, systemic therapies, and tumor biology have transformed CRC care into a multidisciplinary and personalized continuum [1, 265].

Contemporary management encompasses curative‐intent strategies for early‐stage disease, including endoscopic and surgical approaches, as well as increasingly sophisticated systemic therapies for locally advanced and metastatic CRC. In selected patients, these approaches can achieve long‐term disease control and, in curative‐intent settings, durable remission or cure.

A major paradigm shift is the transition from purely stage‐based treatment to molecularly informed decision‐making. Biomarkers such as RAS, BRAF, HER2, and MSI/dMMR now guide targeted therapy and immunotherapy selection, while primary tumor location (right vs. left) further refines treatment strategies, particularly in anti‐EGFR therapy [206]. Patient‐related factors, including performance status, comorbidities, and treatment goals, also play a critical role in individualized care.

These elements are integrated through multidisciplinary team (MDT) decision‐making, which ensures optimal sequencing and combination of treatment modalities, including surgery, chemotherapy, radiotherapy, targeted therapy, and immunotherapy. In rectal cancer, coordinated use of neoadjuvant therapy and precise surgery has improved local control and functional outcomes [319, 320]. In metastatic disease, biologically targeted therapies—such as anti‐VEGF, anti‐EGFR, BRAF inhibitors, HER2‐directed therapies, and emerging KRAS‐targeted approaches—have expanded treatment options and improved survival [277, 282].

Collectively, CRC management has evolved toward a systems‐level precision oncology model, in which treatment is adapted to tumor biology, disease stage, and patient context. The following sections outline stage‐specific strategies, molecularly guided therapies, and emerging approaches that continue to reshape the therapeutic landscape.

### 8.1 Principles of Multidisciplinary Management

Optimal management of CRC requires a MDT approach that integrates expertise from surgeons, medical oncologists, radiation oncologists, gastroenterologists, radiologists, pathologists, genetic counselors, and supportive care specialists. This collaborative framework is essential for navigating the complexity of CRC, where treatment decisions depend on tumor stage, molecular characteristics, anatomical location, and patient‐specific factors [247, 265, 318].

MDT discussions, typically conducted through structured tumor boards, provide a platform for integrating clinical, imaging, histopathological, and molecular data to develop individualized treatment strategies [321]. Core principles include accurate staging, comprehensive risk stratification, and optimal sequencing of surgery, systemic therapy, and radiotherapy.

This approach is particularly critical in rectal cancer, where coordination between neoadjuvant therapy and surgery is essential for achieving tumor downstaging, negative margins, and organ preservation [319]. MDT management also enables identification of patients eligible for curative‐intent multimodal strategies, such as metastasectomy or conversion therapy in selected metastatic cases.

Increasingly, MDT frameworks incorporate molecular and genetic information, including biomarker‐driven therapy selection and referral for germline testing in hereditary syndromes [322]. Patient‐centered considerations—such as performance status, comorbidities, quality of life, and treatment preferences—are integral to decision‐making.

Beyond treatment planning, MDT care supports continuity across the disease trajectory, including supportive care, survivorship planning, and long‐term follow‐up. Collectively, multidisciplinary management represents a cornerstone of modern CRC care, enabling integration of evolving evidence and diverse expertise to optimize outcomes.

### 8.2 Management of Localized Colon Cancer

#### 8.2.1 Endoscopic Resection for Selected Lesions

For carefully selected patients with early‐stage colorectal neoplasia, particularly carcinoma in situ (Tis) or superficial T1 lesions, endoscopic resection provides a minimally invasive and potentially curative treatment option that avoids surgical morbidity [323, 324]. Techniques such as EMR and ESD enable removal of lesions, with ESD allowing en bloc resection of larger or nonpolypoid lesions and more accurate histopathological assessment. Patient selection is primarily based on the estimated risk of lymph node metastasis, which remains low in lesions with favorable features, including well or moderately differentiated histology, limited submucosal invasion, absence of lymphovascular invasion, and negative margins [325]. Accurate preresection evaluation using high‐definition endoscopy and advanced imaging techniques is essential. Following resection, histopathological assessment determines curative status and the need for additional surgery in the presence of high‐risk features. Although associated with potential complications such as bleeding and perforation, particularly with ESD, endoscopic resection plays a central role in organ‐preserving, stage‐adapted management and CRC prevention within screening programs.

#### 8.2.2 Surgical Resection

Surgical resection remains the cornerstone of curative treatment for localized colon cancer, aiming to achieve complete tumor removal and accurate pathological staging. Standard management involves segmental colectomy with en bloc resection of the tumor and associated mesentery, including regional lymph nodes, with at least 12 nodes recommended for adequate staging [326]. The principle of R0 resection combined with appropriate lymphadenectomy is fundamental, while techniques such as complete mesocolic excision with central vascular ligation further enhance oncologic clearance [327, 328, 329, 330]. Minimally invasive approaches, including laparoscopic and robotic colectomy, are now widely adopted and offer faster recovery and improved postoperative outcomes without compromising oncologic efficacy [331]. Surgical decision‐making increasingly incorporates patient factors, tumor location, and integration with perioperative systemic therapy. In emergency settings, procedures may require adaptation, including staged approaches [332]. Overall, surgical outcomes are strongly influenced by technical expertise and institutional quality, underscoring the importance of MDT‐based care in high‐volume centers [333, 334].

#### 8.2.3 Adjuvant Chemotherapy

Adjuvant chemotherapy is a key component of curative‐intent treatment, targeting micrometastatic disease to reduce recurrence risk and improve survival [335, 336]. It is standard of care for Stage III disease and is recommended for selected high‐risk Stage II patients with adverse features such as T4 tumors, lymphovascular invasion, poor differentiation, obstruction or perforation, and inadequate nodal evaluation. Treatment is based on fluoropyrimidine regimens, including 5‐FU/leucovorin or capecitabine, combined with oxaliplatin in FOLFOX or CAPOX, which improve survival compared with monotherapy. Recent efforts focus on risk‐adapted duration to balance efficacy and toxicity, with shorter courses appropriate for selected low‐risk patients. Emerging strategies incorporate molecular biomarkers, particularly ctDNA, to refine recurrence risk and identify patients who may warrant closer consideration of adjuvant therapy; however, in routine practice ctDNA remains complementary to clinicopathological assessment, and its treatment‐guiding role differs between Stage II and Stage III disease [34].

Randomized evidence regarding ctDNA‐informed adjuvant therapy is context‐specific. In Stage II colon cancer, the DYNAMIC trial showed that a ctDNA‐guided strategy reduced chemotherapy use while maintaining similar 5‐year recurrence‐free and overall survival compared with standard management [33]. However, DYNAMIC‐III did not establish noninferiority for ctDNA‐guided treatment de‐escalation in Stage III colon cancer, and treatment escalation in ctDNA‐positive patients did not improve recurrence‐free survival [34]. Accordingly, ctDNA may inform treatment discussions in carefully selected Stage II settings, but it should not routinely replace established clinicopathological risk assessment or standard adjuvant treatment in Stage III disease outside prospective protocols.

### 8.3 Management of Rectal Cancer

#### 8.3.1 Neoadjuvant Chemoradiotherapy/Total Neoadjuvant Therapy

For patients with locally advanced rectal cancer (typically cT3–T4 or node‐positive disease), neoadjuvant therapy is a central component of management, aiming to improve local control and facilitate curative resection. Conventional treatment involves long‐course chemoradiotherapy using fluoropyrimidine‐based chemotherapy combined with pelvic radiation to achieve tumor downstaging, increase the likelihood of negative margins, and reduce local recurrence. More recently, treatment has shifted toward total neoadjuvant therapy (TNT), which incorporates systemic chemotherapy before or after chemoradiotherapy prior to surgery, addressing both local disease and occult micrometastases [265, 320]. TNT is associated with higher rates of pathological complete response, improved treatment compliance, and earlier delivery of systemic therapy, and it increases opportunities for organ preservation strategies in selected patients [337, 338]. Despite these advantages, optimal sequencing and regimen selection remain under investigation, with treatment decisions increasingly guided by MRI staging, risk features such as threatened circumferential resection margin, and patient factors. Overall, TNT represents a major paradigm shift toward integrated, multimodal, and personalized management.

#### 8.3.2 Surgery

Surgical resection remains the cornerstone of curative treatment for rectal cancer, with total mesorectal excision established as the gold standard for achieving optimal oncological outcomes [265]. Total mesorectal excision surgery involves precise dissection along embryological planes to remove the rectum and mesorectal envelope intact, thereby reducing local recurrence through complete clearance of microscopic disease. Surgical approach is determined by tumor location, stage, and response to neoadjuvant therapy, ranging from low anterior resection for mid‐ and upper‐rectal tumors to abdominoperineal resection for low tumors involving the sphincter complex. A key objective in modern surgery is sphincter preservation and avoidance of permanent stoma when oncologically safe, supported by advances in minimally invasive techniques, including laparoscopic and robotic total mesorectal excision surgery [339, 340]. Minimally invasive approaches can improve selected perioperative outcomes, but consistent superiority of robotic over laparoscopic surgery for long‐term urinary, sexual, or oncological outcomes has not been definitively established [340]. Surgical decision‐making is increasingly integrated within MDT frameworks and incorporates response to neoadjuvant therapy, enabling more tailored and function‐preserving strategies.

#### 8.3.3 Organ Preservation Strategies

Organ preservation strategies have emerged as an important paradigm shift for selected patients with rectal cancer who achieve a complete clinical response after neoadjuvant therapy. The watch‐and‐wait approach defers radical surgery in favor of intensive surveillance, aiming to avoid the morbidity associated with total mesorectal excision, including permanent stoma and long‐term functional impairment [341]. Careful patient selection is essential and relies on clinical examination, endoscopy, and high‐resolution MRI to confirm complete response [342, 343]. While this strategy offers substantial quality‐of‐life benefits, it carries a risk of local regrowth, most commonly within the first 2 years; however, early detection through structured surveillance allows effective salvage surgery in most cases [344]. In current practice, this approach is most defensible in experienced multidisciplinary centers with rigorous surveillance infrastructure rather than as a universally transferable pathway [345].

### 8.4 Systemic Therapy for Advanced and Metastatic CRC

#### 8.4.1 Resectable and Potentially Resectable Metastatic Disease

Patients with liver‐limited, lung‐limited, or otherwise oligometastatic CRC should be evaluated by a MDT for curative‐intent resection or local ablative treatment. Conversion systemic therapy may be considered for initially unresectable but potentially convertible disease, followed by reassessment for surgery, thermal ablation, or stereotactic radiotherapy. Selection should integrate technical resectability, extrahepatic disease, tumor biology, molecular profile, response to systemic therapy, patient fitness, and expected preservation of organ function. These strategies apply to highly selected patients and should not be generalized to all metastatic disease [318].

For highly selected patients with limited colorectal peritoneal metastases and the potential for complete cytoreduction, cytoreductive surgery may be considered in experienced centers. The PRODIGE 7 trial did not demonstrate an overall survival benefit from adding short‐duration oxaliplatin‐based HIPEC to cytoreductive surgery [346]; therefore, HIPEC should not be presented as a routine component of treatment for all patients with colorectal peritoneal metastases. Its role remains regimen‐, center‐, and patient‐selection‐dependent.

#### 8.4.2 Standard Chemotherapy Backbones

Systemic chemotherapy remains the foundation of treatment for mCRC and serves as the platform upon which targeted therapies and immunotherapy are integrated. Standard regimens are based on fluoropyrimidines combined with oxaliplatin (FOLFOX, CAPOX) or irinotecan (FOLFIRI), which demonstrate comparable efficacy and are selected according to patient performance status, comorbidities, tumor burden, and treatment goals [247, 318]. In clinical practice, sequential strategies are commonly employed to expose patients to multiple active agents while minimizing cumulative toxicity, whereas more intensive regimens such as FOLFOXIRI may be used in fit patients requiring rapid tumor shrinkage, particularly for potentially resectable disease [110, 347]. Maintenance strategies are also widely adopted to balance efficacy and tolerability after initial disease control [318, 347]. Chemotherapy is frequently combined with targeted agents, enhancing efficacy but also introducing toxicity considerations, including oxaliplatin‐induced neuropathy and irinotecan‐related diarrhea. Overall, chemotherapy remains a versatile and essential backbone in the dynamic, adaptive management of mCRC.

#### 8.4.3 Anti‐VEGF Strategies

Antiangiogenic therapy targeting the VEGF pathway represents a central component of mCRC treatment, with bevacizumab widely used in combination with chemotherapy across multiple lines of therapy [348, 349]. By inhibiting VEGF signaling, these agents normalize tumor vasculature, reduce angiogenesis, and enhance delivery of cytotoxic drugs [350]. Additional agents, including aflibercept and ramucirumab, extend this strategy into later‐line settings following progression [351]. A major advantage of anti‐VEGF therapy is its broad applicability across molecular subtypes, but this also reflects a key limitation: clinically useful predictive biomarkers remain lacking [352]. Benefits are generally modest and accompanied by characteristic toxicities such as hypertension, proteinuria, bleeding, and thromboembolic events [347, 353, 354]. Thus, anti‐VEGF therapy is best understood as a broadly deployable backbone strategy rather than a highly biomarker‐selected precision intervention. In refractory mCRC, later‐line nonbiomarker‐restricted options have also evolved. Trifluridine/tipiracil plus bevacizumab and the oral VEGFR inhibitor fruquintinib provide additional options for previously treated patients, although benefits remain modest and patient selection should consider performance status, prior toxicity, disease tempo, and treatment goals [16].

#### 8.4.4 Anti‐EGFR Strategies

Anti‐EGFR therapy represents a key precision medicine approach in mCRC, with cetuximab and panitumumab demonstrating significant benefit in selected patients [109, 355]. Efficacy is restricted to RAS wild‐type tumors, as activating KRAS or NRAS mutations lead to constitutive downstream signaling and resistance to EGFR inhibition, making molecular testing mandatory prior to treatment. Primary tumor location also matters, with left‐sided tumors showing greater responsiveness than right‐sided disease. In clinical practice, anti‐EGFR agents are most useful in RAS wild type, predominantly left‐sided mCRC, particularly when tumor shrinkage is a priority. Resistance remains a major challenge, arising through secondary RAS mutations or activation of alternative pathways, which can increasingly be tracked with ctDNA monitoring [278, 279].

#### 8.4.5 BRAF‐Targeted Approaches

BRAF V600E‐mutant mCRC represents an aggressive molecular subtype characterized by constitutive MAPK pathway activation and poor prognosis. Single‐agent BRAF inhibition is ineffective in CRC because EGFR feedback rapidly restores downstream signaling, necessitating combination approaches. Dual targeting of BRAF and EGFR, with or without MEK inhibition, has improved response rates and survival compared with conventional therapy and is established in previously treated patients. More recently, earlier‐line intensification strategies have begun to reshape care in this subgroup, but treatment remains relevant to a narrowly defined molecular population rather than to CRC broadly [310].

Clinical development in BRAF V600E‐mutant mCRC has advanced rapidly. In the Phase 3 BREAKWATER trial, first‐line encorafenib plus cetuximab and mFOLFOX6 improved objective response, progression‐free survival, and overall survival compared with standard care in patients with previously untreated BRAF V600E‐mutant mCRC [39]. These data support BRAF/EGFR‐directed intensification as an important biomarker‐defined strategy for selected patients with BRAF V600E‐mutant mCRC. In the United States, encorafenib in combination with cetuximab and fluorouracil‐based chemotherapy has received FDA approval for adult patients with BRAF V600E‐mutant mCRC detected by an FDA‐authorized test, while the strategy remains confined to a narrowly selected molecular subgroup [311].

#### 8.4.6 HER2‐Targeted Approaches

HER2‐positive CRC, defined by ERBB2 amplification or overexpression, represents a distinct molecular subgroup, particularly in RAS/BRAF wild‐type tumors resistant to anti‐EGFR therapy. HER2 activation promotes tumor growth through MAPK and PI3K/AKT pathways and contributes to therapeutic resistance. Clinical trials have demonstrated that HER2‐directed strategies—including dual HER2 blockade with monoclonal antibodies or tyrosine kinase inhibitors, as well as antibody–drug conjugates—can achieve meaningful responses in refractory mCRC [40, 280, 356]. At present, the main clinical role of HER2‐targeted therapy is in selected later‐line, biomarker‐defined populations rather than in broad routine use across CRC.

HER2‐directed therapy has also diversified [41, 42]. Results from DESTINY‐CRC02 confirmed clinically meaningful activity of trastuzumab deruxtecan in heavily pretreated HER2‐positive advanced CRC while emphasizing dose optimization and risk‐aware toxicity management [40]. Complementing antibody–drug conjugate‐based development, the final analysis of MOUNTAINEER supported tucatinib plus trastuzumab as an active chemotherapy‐refractory option in HER2‐positive, RAS wild‐type metastatic CRC, further broadening the therapeutic landscape of HER2‐directed therapy in this molecular subset [42]. Biomarker analyses from DESTINY‐CRC01 additionally suggest that HER2‐related biomarkers may help refine selection for trastuzumab deruxtecan in HER2‐expressing metastatic CRC [41]. In the United States, tucatinib plus trastuzumab has received FDA accelerated approval for adults with RAS wild type, HER2‐positive unresectable or metastatic CRC that has progressed after fluoropyrimidine‐, oxaliplatin‐, and irinotecan‐based chemotherapy [357].

#### 8.4.7 KRAS G12C and Other Emerging Targets

The therapeutic landscape of mCRC is expanding toward a highly stratified, biomarker‐driven model that includes emerging targets such as KRAS G12C. Although RAS mutations were historically considered undruggable, mutation‐specific inhibitors have demonstrated activity, particularly when paired with EGFR blockade to suppress adaptive feedback signaling [43]. More broadly, the treatment paradigm now includes EGFR‐, VEGF‐, BRAF‐, and HER2‐directed therapies, with additional tumor‐agnostic targets such as NTRK fusions further extending options in selected patients. The key point is not that mCRC has become universally targetable, but that actionable therapy is increasingly organized around narrowly defined molecular subsets.

KRAS G12C is a clear example of how resistance biology can inform rational combination therapy. In KRYSTAL‐1, adagrasib plus cetuximab produced an objective response rate of 34.0%, with a median duration of response of 5.8 months [43], supporting its clinical use in previously treated KRAS G12C‐mutated metastatic CRC. Similarly, in CodeBreaK 300, sotorasib plus panitumumab achieved a median progression‐free survival of 5.6 months compared with 2.2 months with investigator‐selected standard care, with objective response rates of 26% and 0%, respectively [44]. These results support sotorasib plus panitumumab as an evidence‐based later‐line option for previously treated KRAS G12C‐mutated metastatic CRC. Together, these data are clinically important but apply to a small molecular subset and do not eliminate the broader challenge of pathway plasticity and acquired resistance. These trial results have been accompanied by US FDA approvals of EGFR‐combined KRAS G12C inhibitor regimens in previously treated disease: adagrasib plus cetuximab for KRAS G12C‐mutated locally advanced or metastatic CRC after prior fluoropyrimidine‐, oxaliplatin‐, and irinotecan‐based chemotherapy, and sotorasib plus panitumumab for KRAS G12C‐mutated mCRC after the same prior chemotherapy exposure [308, 309].

### 8.5 Immunotherapy in CRC

#### 8.5.1 MSI‐H/dMMR CRC

Immune checkpoint inhibitors have demonstrated remarkable efficacy in MSI‐H/dMMR CRC, establishing this subgroup as the most responsive population to immunotherapy in gastrointestinal oncology. In metastatic disease, PD‐1‐based therapy is an established standard for this biomarker‐defined subgroup, and in localized disease neoadjuvant checkpoint blockade has opened the possibility of organ preservation in selected patients [312, 313]. Even within MSI‐H/dMMR CRC, however, not all patients derive durable benefit, underscoring the need for more refined predictive biomarkers and careful implementation [36].

The Phase 3 CheckMate 8HW trial further established nivolumab plus ipilimumab as an important first‐line immunotherapy option for MSI‐H/dMMR metastatic CRC, although treatment choice should still consider disease burden, disease tempo, toxicity tolerance, prior therapy, patient preference, and local regulatory context [313].

#### 8.5.2 Mechanisms of Resistance

Resistance to ICIs in CRC arises from a complex interplay of tumor‐intrinsic, microenvironmental, and systemic factors. Immune exclusion is a key mechanism, often driven by Wnt/β‐catenin or TGF‐β signaling, which prevents effective infiltration of cytotoxic T cells into the tumor core. Defective antigen presentation further limits immune recognition, through alterations in MHC Class I expression, β2‐microglobulin, or interferon signaling pathways. In addition, the TME is frequently immunosuppressive, characterized by regulatory T cells, myeloid‐derived suppressor cells, tumor‐associated macrophages, and inhibitory cytokines such as TGF‐β and IL‐10 [20, 174]. Upregulation of alternative immune checkpoints—including LAG‐3, TIM‐3, and TIGIT—and metabolic constraints such as hypoxia further contribute to immune dysfunction. These mechanisms underscore that resistance is driven by interconnected biological networks, providing the rationale for combination strategies that integrate ICIs with targeted therapy, antiangiogenic agents, radiotherapy, or microenvironment modulation [358, 359].

#### 8.5.3 The Challenge of MSS CRC

MSS CRC, which accounts for the majority of cases, remains largely refractory to immunotherapy and represents a major unmet clinical need [174]. Compared with MSI‐H tumors, MSS CRC typically exhibits lower tumor mutational burden, reduced neoantigen load, and limited immune infiltration, often presenting as immune‐desert or immune‐excluded phenotypes. Combination strategies involving antiangiogenic agents, radiotherapy, chemotherapy, or pathway‐targeted drugs are therefore under active study, but most remain investigational and have not yet produced broadly practice‐defining results [359].

#### 8.5.4 Organ‐Preserving Immunotherapy in Localized dMMR Disease

The most practice‐changing recent advance in localized CRC has been the demonstration that some dMMR tumors can be managed nonoperatively after checkpoint blockade. In the 2025 report, all 49 patients with locally advanced dMMR rectal cancer who completed dostarlimab treatment achieved a clinical complete response and elected nonoperative management. Across the rectal and nonrectal cohorts, 84 of 103 patients who completed treatment achieved a clinical complete response and 82 avoided surgery. In the overall study population, the reported 2‐year recurrence‐free survival was 92% [37].

A related but clinically distinct development is neoadjuvant immunotherapy for localized dMMR colon cancer. In NICHE‐2, 109 of 111 patients in the efficacy analysis achieved a pathological response after short‐course nivolumab plus ipilimumab, including a high pathological complete response rate. These results demonstrate substantial neoadjuvant activity but should not be conflated with the nonoperative rectal‐cancer strategy, because planned surgery remained part of the NICHE‐2 treatment pathway [38].

These data do not eliminate the need for rigorous surveillance, expert radiologic and endoscopic assessment, or careful multidisciplinary selection [36]. Their current significance is best viewed as practice‐changing in selected expert settings rather than as a universally standardized pathway that can be adopted without infrastructure, experience, and close follow‐up.

### 8.6 Supportive Care, Survivorship, and Follow‐Up

#### 8.6.1 Surveillance After Curative Treatment

Post‐treatment surveillance following curative‐intent therapy for CRC is essential for early detection of recurrence, identification of metachronous neoplasms, and optimization of long‐term survivorship care [360, 361]. Standard strategies integrate clinical assessment, carcinoembryonic antigen (CEA) monitoring, cross‐sectional imaging, and periodic colonoscopy, with the most intensive follow‐up occurring within the first 3–5 years when recurrence risk is highest. CEA provides a noninvasive tool for monitoring, although its interpretation requires correlation with imaging due to limited specificity. Imaging is critical for detecting asymptomatic metastases, particularly in the liver and lungs, where early intervention may be curative, while colonoscopy enables detection of local recurrence and new neoplastic lesions. Emerging approaches, including ctDNA‐based monitoring can identify molecular recurrence before conventional radiological detection and may refine recurrence‐risk assessment [314, 315]. However, the ALTAIR trial did not demonstrate a statistically significant disease‐free survival benefit from initiating trifluridine/tipiracil solely on the basis of ctDNA positivity in patients without radiological disease [35]. Therefore, ctDNA‐triggered systemic treatment during postoperative surveillance remains investigational, and conventional clinical assessment, imaging, CEA monitoring, and colonoscopy remain the foundations of follow‐up.

#### 8.6.2 Management of Treatment‐Related Toxicities

Management of treatment‐related toxicities is a critical component of CRC care, ensuring treatment adherence, maintenance of dose intensity, and preservation of quality of life. Toxicities arise from multimodal therapies, including chemotherapy, targeted therapy, immunotherapy, and radiotherapy, each with distinct adverse event profiles. Common chemotherapy‐related toxicities include oxaliplatin‐induced peripheral neuropathy and irinotecan‐associated diarrhea, both of which require proactive management and dose adjustment strategies [362, 363, 364]. Hematologic toxicities such as neutropenia and anemia are also frequent and may necessitate supportive interventions, including growth factors or transfusion. Targeted therapies introduce additional toxicities, including hypertension and proteinuria with anti‐VEGF agents and dermatologic effects with anti‐EGFR therapies, often requiring multidisciplinary management [365, 366]. Immune checkpoint inhibitors may cause immune‐related adverse events affecting multiple organ systems, necessitating early recognition and immunosuppressive treatment when indicated [367]. Beyond physical toxicities, fatigue, nutritional compromise, and psychosocial stress significantly affect patient well‐being, underscoring the importance of comprehensive supportive care [368, 369]. Increasingly, treatment strategies incorporate dose optimization, treatment breaks, and maintenance approaches to balance efficacy and tolerability, particularly in long‐term disease control settings [370].

#### 8.6.3 Nutritional and Lifestyle Interventions

Nutritional and lifestyle interventions are integral to CRC survivorship and can improve physical function, symptom control, and quality of life. Higher physical activity, healthier dietary patterns, and maintenance of metabolic health have also been associated with more favorable oncological outcomes, although causal effects on recurrence and overall survival remain incompletely established for many interventions [369, 371]. Patients frequently experience nutritional challenges, including weight loss, sarcopenia, and altered bowel function, necessitating individualized dietary assessment and support focused on maintaining adequate caloric and protein intake while managing treatment‐related symptoms [372, 373]. Lifestyle factors, including diet quality, physical activity, and weight management, are important modifiable determinants of long‐term outcomes, with diets rich in fiber and plant‐based foods, regular physical activity, and maintenance of healthy body weight associated with improved clinical outcomes. These interventions may exert biological effects through modulation of metabolism, systemic inflammation, insulin signaling, and the gut microbiota [87]. Smoking cessation and moderation of alcohol intake further contribute to risk reduction [68]. Despite growing evidence, implementation remains variable, highlighting the need for structured survivorship programs and multidisciplinary support [374, 375]. Overall, nutritional and lifestyle optimization represents a low‐risk, high‐impact strategy that complements medical treatment and supports long‐term CRC care.

Management of CRC therefore depends on aligning treatment intensity, modality sequence, and biologic targeting with the point each patient occupies along the disease continuum [376]. Surgery remains fundamental, but durable benefit increasingly depends on early biomarker testing, rational systemic combinations, thoughtful survivorship planning, and multidisciplinary coordination. Just as importantly, routine standards of care must be distinguished from selectively adopted innovations and from investigational strategies whose biological rationale is strong but whose implementation is not yet mature.

## 9 Preclinical Models and Translational Development of Emerging Therapies in CRC

### 9.1 Why Translational Research Matters in CRC

Translational research matters in CRC because many of the field's most important clinical problems—immune resistance in MSS disease, pathway escape after targeted therapy, uncertainty around biomarker implementation, and the gap between anatomical stage and biological behavior—cannot be solved by empiric treatment layering alone [1, 201].

The value of translational research is therefore not simply to generate more models, but to determine which mechanisms are actionable, which biomarkers are clinically informative, and which combinations are sufficiently justified to move into biomarker‐linked trials and adaptive care pathways [377, 378].

### 9.2 Preclinical Models Used in CRC Research

#### 9.2.1 Genetically Engineered Mouse Models

Genetically engineered mouse models (GEMMs) are fundamental tools for studying CRC tumorigenesis, enabling controlled manipulation of key driver genes such as APC, KRAS, TP53, and SMAD4. Classic models, including Apc^Min/+^, have provided key insights into Wnt/β‐catenin‐driven tumor initiation, while compound models incorporating multiple mutations more closely recapitulate the adenoma–carcinoma sequence and progression to invasive disease [379, 380]. A major advantage of GEMMs is their ability to model CRC within an intact immune system, allowing investigation of tumor–host interactions, immune regulation, and response to immunotherapy. These models are also valuable for studying early tumorigenic events and prevention strategies. However, limitations include long latency, limited heterogeneity compared with human tumors, and difficulty in modeling advanced metastasis. Differences between murine and human biology further constrain direct translation [381]. Despite these challenges, GEMMs remain indispensable for mechanistic studies and therapeutic development when integrated with complementary model systems.

#### 9.2.2 AOM/DSS Inflammation‐Associated Models

The AOM/DSS model is widely used to study inflammation‐associated CRC, closely mimicking colitis‐associated cancer in IBD. In this system, AOM induces mutagenic DNA damage, while repeated DSS exposure causes epithelial injury and chronic inflammation, together driving tumorigenesis [379, 382]. This model is particularly valuable for dissecting the role of inflammatory signaling pathways, including NF‐κB and STAT3, as well as cytokine networks and immune cell infiltration in tumor development. It also provides an important platform for studying host–microbiota interactions, as barrier disruption allows direct interaction between microbial products and the mucosa. However, the model does not fully recapitulate the genetic landscape of sporadic CRC, as tumorigenesis is driven primarily by inflammation rather than sequential oncogenic mutations, and variability in DSS‐induced colitis can affect reproducibility [383]. Nevertheless, AOM/DSS remains highly relevant for investigating inflammation‐driven tumorigenesis and microbiota‐related mechanisms.

#### 9.2.3 Xenograft and Patient‐Derived Xenograft Models

Xenograft models, including cell line‐derived xenografts (CDX) and patient‐derived xenografts (PDX), are widely used for drug testing and translational research in CRC. CDX models allow rapid and reproducible evaluation of therapeutic efficacy but lack the genetic diversity and structural complexity of primary tumors. In contrast, PDX models preserve tumor architecture, genomic features, and intratumoral heterogeneity, making them highly valuable for studying treatment response, resistance mechanisms, and tumor evolution. These models have also been explored as personalized “avatar” systems to guide therapy selection [384, 385]. However, their use is limited by the absence of a functional human immune system in most models, restricting immunotherapy studies, as well as by high cost, variable engraftment, and potential selection bias. Humanized mouse models partially address these limitations but remain technically challenging [380, 386]. Overall, xenograft systems are essential for bridging preclinical findings with clinical application.

#### 9.2.4 Organoids and Coculture Systems

Organoid models derived from patient tumors represent highly versatile in vitro platforms that preserve key genetic, transcriptomic, and structural features of CRC [18, 387, 388]. Compared with traditional cell lines, organoids more accurately capture tumor heterogeneity and cellular organization, making them valuable for studying tumor biology, signaling pathways, and drug response. Patient‐derived organoids enable high‐throughput drug screening and hold promise as personalized medicine tools for predicting therapeutic response. Integration with coculture systems further enhances physiological relevance by incorporating components of the TME, including immune cells, fibroblasts, endothelial cells, and microbiota‐related factors, thereby enabling investigation of TME interactions and immunotherapy responses [389, 390]. Despite these advantages, challenges remain in standardization, variability in culture conditions, and incomplete representation of systemic biology. Nonetheless, organoid‐based systems represent a major advancement in translational CRC research, bridging the gap between in vitro and in vivo models and supporting precision oncology.

#### 9.2.5 Spatial Multiomics and Organoid 2.0 Platforms

A rapidly growing frontier is the integration of spatial transcriptomics, single‐cell sequencing, and advanced organoid platforms. Spatial atlases now allow investigators to map epithelial programs, fibroblast states, immune exclusion patterns, and invasion‐related niches within intact CRC tissues, thereby linking cell‐state heterogeneity to anatomy and treatment resistance [115].

At the same time, next‐generation organoid systems are moving beyond tumor epithelium alone to incorporate immune and stromal components, enabling more realistic modeling of therapy response, immune editing, and fibroblast‐mediated resistance. These hybrid platforms may become especially valuable for testing combination therapies in molecularly and microenvironmentally defined CRC subsets [391, 392].

### 9.3 Preclinical Studies Targeting Oncogenic Pathways

#### 9.3.1 Wnt/β‐Catenin‐Directed Strategies

Wnt/β‐catenin signaling plays a central role in CRC initiation, most commonly driven by APC loss‐of‐function mutations, making it a biologically compelling but clinically challenging therapeutic target. Preclinical studies have explored multiple intervention points within this pathway, including inhibition of β‐catenin–mediated transcription, targeting upstream regulators such as PORCN, and disrupting interactions between β‐catenin and transcriptional coactivators. These approaches have demonstrated the ability to suppress tumor growth, reduce stem‐like tumor‐cell states, and impair tumor‐initiating capacity [4, 393, 394]. In addition, Wnt inhibition can modulate the TME, enhancing immune infiltration and providing a rationale for combination with immunotherapy. However, clinical translation remains challenging due to significant on‐target toxicity in normal tissues with high Wnt activity, as well as pathway complexity, redundancy, and extensive crosstalk with MAPK, PI3K, and TGF‐β signaling [20]. As a result, current strategies increasingly focus on context‐specific modulation and rational combination approaches to balance efficacy and safety.

#### 9.3.2 MAPK and PI3K Pathway Inhibition

The MAPK (RAS–RAF–MEK–ERK) and PI3K/AKT/mTOR pathways are central regulators of proliferation, survival, and therapeutic resistance in CRC, particularly in tumors harboring RAS or BRAF mutations [116, 199, 395]. These pathways are frequently coactivated and exhibit extensive crosstalk, enabling tumors to maintain oncogenic signaling despite single‐agent inhibition. Preclinical studies have shown that targeted inhibition often triggers adaptive feedback mechanisms, including upregulation of receptor tyrosine kinases and activation of parallel pathways, thereby limiting efficacy. Consequently, combination strategies targeting multiple nodes—such as dual BRAF and EGFR inhibition or combined MAPK and PI3K blockade—have demonstrated enhanced antitumor activity and delayed resistance in experimental models. These findings have informed current clinical approaches in molecularly defined subgroups [396]. However, challenges remain due to toxicity, limited therapeutic windows, and interpatient variability in pathway dependence. Overall, these studies highlight the need for network‐based therapeutic strategies that anticipate and overcome adaptive resistance mechanisms.

#### 9.3.3 EMT, Metastasis, and TME‐Oriented Interventions

Preclinical models have provided critical insights into epithelial–mesenchymal transition (EMT), metastatic dissemination, and TME remodeling, all of which contribute to CRC progression and therapeutic resistance. EMT enables tumor cells to acquire invasive and stem cell–like properties through coordinated regulation by pathways such as TGF‐β, Wnt/β‐catenin, Notch, and MAPK, along with transcription factors including SNAIL, ZEB, and TWIST [20, 397]. Beyond tumor‐intrinsic mechanisms, the TME—comprising cancer‐associated fibroblasts, immune cells, and extracellular matrix components—actively promotes invasion, angiogenesis, and immune evasion. Preclinical studies have demonstrated that targeting stromal and immune components, such as inhibition of TGF‐β signaling, angiogenesis pathways, or immunosuppressive cell populations, can disrupt protumorigenic interactions and enhance therapeutic response [122]. Importantly, metastasis is increasingly recognized as a systems‐level process driven by dynamic tumor–host interactions, including formation of premetastatic niches [398]. However, clinical translation remains challenging due to TME heterogeneity and adaptability, as well as potential off‐target effects. These findings support the development of combination strategies targeting both tumor‐intrinsic pathways and the surrounding microenvironment.

### 9.4 Preclinical and Translational Studies on Microbiota Modulation

The gut microbiota has emerged as a potentially modifiable regulator of CRC biology, influencing tumor initiation, progression, and therapeutic response through interactions with host metabolism, immune signaling, and oncogenic pathways [59, 87]. Preclinical studies implicate pathogenic taxa such as *Fusobacterium nucleatum*, enterotoxigenic *Bacteroides fragilis*, and pks‐positive *Escherichia coli* in inflammatory signaling and genotoxic stress, whereas beneficial commensals and short‐chain fatty acids may support epithelial integrity and immune surveillance [176, 399]. These insights have motivated microbiota‐directed strategies including dietary modulation, probiotics, prebiotics, antibiotics, and fecal microbiota transplantation. At present, however, clinical translation remains limited by interindividual variability, incomplete mechanistic resolution, and the absence of standardized intervention frameworks [400, 401].

Microbiota‐targeted translation remains promising but methodologically demanding. Future progress will require standardized sampling, functional validation, and intervention studies capable of separating causal immune or metabolic effects from confounding by diet, transit time, inflammation, or treatment exposure [89, 194].

### 9.5 Clinical Trials of Targeted Therapy and Immunotherapy

#### 9.5.1 EGFR/VEGF/BRAF/HER2‐Directed Trials

Clinical trials targeting oncogenic signaling pathways have established a biomarker‐driven therapeutic framework in mCRC, integrating targeted agents with chemotherapy to improve outcomes. Anti‐VEGF therapy, such as bevacizumab, has demonstrated consistent benefit across treatment lines regardless of molecular subtype, while anti‐EGFR antibodies (cetuximab, panitumumab) provide substantial benefit in RAS wild‐type tumors, particularly in left‐sided disease, highlighting the importance of molecular and anatomical stratification [402]. For poor‐prognosis subgroups, such as BRAF V600E‐mutant CRC, combination strategies targeting BRAF and EGFR have significantly improved outcomes, reflecting the need to overcome pathway reactivation [39]. Similarly, HER2‐targeted therapies have shown meaningful activity in HER2‐amplified, RAS/BRAF wild‐type CRC, particularly in refractory settings [42]. Collectively, these trials illustrate the translation of preclinical insights into mechanism‐based combination strategies and underscore the central role of molecular profiling in guiding treatment selection.

#### 9.5.2 Immune Checkpoint Blockade Trials

Clinical trials of immune checkpoint inhibitors have established MSI‐H/dMMR CRC as a highly responsive subgroup, with PD‐1 blockade demonstrating durable responses, prolonged survival, and in some cases long‐term remission compared with conventional chemotherapy. These benefits reflect the high mutational burden and immunogenic microenvironment characteristic of MSI‐H tumors [312, 403]. However, not all patients respond, and resistance remains a significant challenge, prompting investigation of combination strategies including dual checkpoint blockade and integration with chemotherapy, targeted therapy, antiangiogenic agents, radiotherapy, and TME modulation [174]. In contrast, MSS CRC shows limited response to monotherapy, leading to extensive trials aimed at overcoming immune resistance through strategies that enhance antigen presentation, improve immune infiltration, and reverse immune suppression [359, 404]. These studies collectively define a translational framework linking tumor immunobiology with therapeutic innovation.

#### 9.5.3 MSS CRC Combination Strategies

Given the intrinsic resistance of MSS CRC to immunotherapy, current clinical research focuses on combination strategies designed to increase antigen release, improve immune‐cell infiltration, and mitigate immune exclusion in selected MSS tumors. MSS tumors are characterized by low tumor mutational burden, limited neoantigen load, and an immunosuppressive microenvironment with sparse T‐cell infiltration. To overcome these barriers, trials are evaluating combinations of immune checkpoint inhibitors with antiangiogenic agents, targeted therapies, chemotherapy, and radiotherapy. Anti‐VEGF therapy may enhance immune cell infiltration by normalizing tumor vasculature, while MAPK or TGF‐β pathway inhibition can reverse immune exclusion [404]. Chemotherapy can induce immunogenic cell death, increasing antigen release and immune activation, whereas radiotherapy may promote in situ vaccination‐like immune effects. Emerging approaches also include microbiota modulation and novel immunotherapies. Although results remain modest, these strategies reflect a shift toward mechanism‐based combinatorial approaches guided by tumor biology and immune context.

#### 9.5.4 KRAS G12C and Other New Targets

Clinical trials targeting KRAS G12C and other rare genomic alterations are expanding therapeutic options in mCRC and highlight the growing importance of comprehensive molecular profiling. KRAS G12C inhibitors represent a breakthrough in targeting previously “undruggable” mutations, although responses in CRC are limited due to rapid feedback activation of EGFR signaling, prompting combination strategies with anti‐EGFR therapy [405]. Beyond KRAS G12C, therapies targeting rare alterations such as NTRK fusions, RET or ALK rearrangements, and other tumor‐agnostic targets are being evaluated, collectively defining a clinically meaningful subset of patients eligible for precision therapies [316, 317]. These developments reflect a shift from empiric treatment to a highly segmented, biomarker‐driven model, in which comprehensive genomic profiling enables identification of actionable alterations and optimization of therapeutic strategies in advanced disease.

#### 9.5.5 CAR‐T/CAR‐NK and Other Adoptive Cellular Immunotherapies

Adoptive cellular immunotherapy has emerged as a potentially relevant but still highly investigational strategy in CRC, particularly in microsatellite‐stable disease, where conventional immune checkpoint blockade remains ineffective for most patients. Compared with hematologic malignancies, translation into CRC is constrained by heterogeneous antigen expression, limited trafficking and persistence within tumor tissue, stromal and vascular barriers, and a profoundly immunosuppressive microenvironment [406]. At present, CAR‐T/CAR‐NK therapy should be regarded as proof‐of‐concept rather than as a component of routine CRC care.

One of the most notable recent clinical signals has come from an early‐phase trial of GCC19CART in heavily pretreated metastatic CRC, which showed objective responses and manageable but nontrivial toxicity [407]. Early CAR‐NK experience has similarly suggested feasibility and acceptable safety but limited efficacy [408]. Taken together, these findings indicate that adoptive cellular therapy in CRC has progressed from theoretical possibility to early human proof‐of‐concept, but remains far from standard practice [409].

### 9.6 Radiotherapy Combined With Immunotherapy

Radiotherapy has emerged as a promising partner for immunotherapy in CRC by modulating the TME and enhancing antitumor immune responses [410]. Beyond local tumor control, radiotherapy can induce immunogenic cell death, releasing damage‐associated molecular patterns and tumor antigens that promote dendritic‐cell activation and T‐cell priming, thereby promoting in situ vaccination‐like immune effects. Preclinical studies have demonstrated increased T‐cell infiltration, enhanced antigen presentation, and upregulation of immune checkpoint molecules following radiation, thereby sensitizing tumors to immune checkpoint blockade. These effects are particularly relevant in MSS CRC, where radiotherapy may increase antigen release, immune‐cell recruitment, and local inflammatory signaling in selected settings, although reliable conversion of MSS CRC into an immunotherapy‐responsive phenotype has not been established clinically. Early clinical studies have reported signals of enhanced response and potential abscopal effects, although results remain preliminary [411, 412, 413]. Challenges include optimizing radiation dose, fractionation, sequencing with immunotherapy, and patient selection, as well as balancing immune activation with toxicity [414]. Ongoing studies aim to refine these strategies and integrate radiotherapy into multimodal, biomarker‐driven treatment approaches, highlighting its role as a mechanistically attractive but still investigational component of combination immunotherapy strategies. To integrate the translational logic developed across this section, Table 4 reorganizes representative CRC models and therapeutic strategies by biological category, biological rationale, clinical positioning, and major resistance or implementation barriers.

**TABLE 4:** Translational development strategies in colorectal cancer organized by biological category, representative models, clinical positioning, and resistance or implementation barriers.

Biological category	Target/strategy	Representative models/platforms	Key translational insight	Current clinical positioning	Major resistance mechanisms/implementation barriers	References
Pathway‐targeted strategy	APC loss‐driven Wnt/β‐catenin activation	APC‐mutant genetically engineered mouse models, patient‐derived organoids, stromal cocultures	APC loss drives aberrant Wnt/β‐catenin signaling, supporting colorectal tumor initiation and stemness; however, dependence of normal intestinal epithelium on Wnt signaling limits simple monotherapy approaches.	Investigational; mainly preclinical to early‐phase and combination‐oriented development.	On‐target toxicity in normal intestinal epithelium, pathway redundancy, context‐dependent pathway activity, and limited therapeutic window.	[4, 112, 113, 114, 115, 387, 388, 389, 393, 394]
Actionable molecular driver	KRAS G12C–EGFR feedback signaling	KRAS G12C‐mutant cell lines, patient‐derived xenograft models, adaptive signaling studies	Single‐agent KRAS G12C inhibition is limited by EGFR feedback, supporting vertical pathway blockade with EGFR inhibition.	US FDA‐approved later‐line targeted strategy in combination with EGFR blockade for adults with previously treated KRAS G12C‐mutated advanced or metastatic colorectal cancer.	EGFR rebound activation, secondary RAS pathway alterations, compensatory MAPK or PI3K signaling, and acquired pathway bypass.	[43, 44, 308, 309, 405]
	BRAF V600E pathway rebound	BRAF‐mutant xenografts and resistance models	BRAF inhibition alone is insufficient because EGFR feedback restores MAPK signaling, supporting combined BRAF and EGFR blockade.	Established biomarker‐directed strategy in BRAF V600E‐mutant mCRC; in the United States, encorafenib plus cetuximab with fluorouracil‐based chemotherapy is FDA‐approved for adult patients with BRAF V600E‐mutant mCRC detected by an FDA‐authorized test.	MAPK pathway reactivation, EGFR feedback activation, secondary bypass signaling, and clonal selection under treatment pressure.	[39, 280, 310, 311, 355, 396]
	HER2‐amplified signaling	HER2‐positive organoids and xenografts	HER2 amplification identifies an anti‐EGFR‐resistant but therapeutically actionable subgroup.	HER2‐directed therapy is used in selected HER2‐positive, RAS wild type, refractory metastatic colorectal cancer; antibody–drug conjugates broaden clinical relevance in biomarker‐defined settings.	HER2 heterogeneity or loss, downstream MAPK or PI3K activation, acquired resistance to antibody–drug conjugates, and variable biomarker thresholds.	[40, 41, 42, 281, 282, 357]
Immune strategy	MSI‐H/dMMR immune activation	Immunocompetent neoantigen‐rich systems and organoid‐immune coculture	High neoantigen load and inflamed immune contexture explain deep checkpoint responses and organ‐preservation potential.	Established immunotherapy biomarker in metastatic disease; localized dMMR disease supports organ‐preserving immunotherapy in selected expert settings with rigorous surveillance.	Defective antigen presentation, immune checkpoint adaptation, immunosuppressive cytokine signaling, and heterogeneous response despite mismatch repair deficiency.	[36, 37, 38, 160, 161, 162, 312, 313]
	MSS immune resistance and stromal exclusion	Syngeneic immune‐excluded models, fibroblast‐rich cocultures, radiation‐immunotherapy systems	Meaningful response likely requires simultaneous reversal of immune exclusion, angiogenic support, stromal barriers, and low antigenicity.	Active investigation of ICI combinations with VEGF blockade, radiation, TGF‐β inhibition, and MAPK targeting; not routine care.	Low neoantigen burden, stromal exclusion, myeloid suppression, TGF‐β‐driven stromal and myeloid immune exclusion, limited T‐cell infiltration, and adaptive immunosuppression.	[111, 122, 168, 174, 358, 359, 404, 410, 411, 412, 413, 414]
Emerging therapeutic platform	Adoptive cellular immunotherapy	GCC‐targeted CAR‐T systems, CEA‐targeted CAR platforms, NKG2D‐based CAR‐NK cells, logic‐gated or dual‐target engineered cellular models	Efficacy depends on antigen specificity, trafficking, persistence, and overcoming the immunosuppressive tumor microenvironment.	Early‐phase proof‐of‐concept only; not standard care in colorectal cancer.	Heterogeneous antigen expression, limited tumor trafficking, poor persistence, stromal barriers, on‐target off‐tumor toxicity, and immunosuppressive microenvironment.	[406, 407, 408, 409]
	Microbiota–immune–metabolic modulation	AOM/DSS models, gnotobiotic mice, fecal microbiota transplantation systems	Microbiota can alter inflammation, immune priming, metabolism, and therapeutic sensitivity, but effects remain context‐dependent.	Translational and early clinical exploration; not standardized for routine therapeutic decision‐making.	Interindividual variability, confounding by diet and inflammation, lack of standardized sampling, uncertain causal taxa or functional signatures, and reproducibility challenges.	[87, 89, 175, 176, 194, 400, 401]
Biomarker‐guided management	ctDNA‐informed postoperative risk stratification and intervention trials	Tumor‐informed serial assays, prospective MRD cohorts, and randomized DYNAMIC, DYNAMIC‐III, and ALTAIR trials	Postoperative ctDNA strongly stratifies recurrence risk, but treatment‐guiding utility depends on disease stage, testing time point, and the intervention evaluated.	Established prognostic biomarker; selected Stage II decision‐support evidence is available, whereas routine Stage III escalation/de‐escalation and treatment at molecular recurrence remain investigational.	Low tumor shedding, false‐negative results, assay and timing variability, spontaneous ctDNA clearance, heterogeneous disease stages, and absence of validated interventions for many ctDNA‐positive settings.	[15, 32, 33, 34, 35, 249, 314, 315]

Abbreviations: AOM/DSS, azoxymethane/dextran sodium sulfate; CAR, chimeric antigen receptor; CEA, carcinoembryonic antigen; CRC, colorectal cancer; ctDNA, circulating tumor DNA; dMMR, deficient mismatch repair; EGFR, epidermal growth factor receptor; FMT, fecal microbiota transplantation; GCC, guanylate cyclase C; GEMMs, genetically engineered mouse models; HER2, human epidermal growth factor receptor 2; ICI, immune checkpoint inhibitor; MAPK, mitogen‐activated protein kinase; mCRC, metastatic colorectal cancer; MRD, molecular residual disease; MSI, microsatellite instability; MSS, microsatellite stable; NK, natural killer; PD‐1, programmed cell death protein 1; PDX, patient‐derived xenograft; TGF‐β, transforming growth factor beta; TME, tumor microenvironment; VEGF, vascular endothelial growth factor.

Taken together, the value of translational research in CRC lies not simply in generating new models or layering additional drugs, but in clarifying why a given strategy succeeds, fails, or benefits only a biologically restricted subgroup [1]. Progress will depend on preclinical systems that preserve tumor ecology more faithfully, clinical trials that prioritize mechanism‐based combinations over empiric escalation, and biomarker frameworks that prospectively connect experimental vulnerability to patient selection and treatment sequencing [415].

## 10 Conclusions and Perspectives

CRC should now be understood as a systems‐level disease rather than a narrowly organ‐confined malignancy. Its population burden is shaped by demographic transition, lifestyle exposures, and unequal health‐system capacity; its biology is governed by interacting genomic, epigenetic, immune, stromal, metabolic, and microbial processes; and its clinical course reflects both inherited susceptibility and adaptive tumor evolution under therapeutic pressure.

Several developments have already begun to alter clinical practice. Organized screening, high‐quality colonoscopy, and risk‐adapted follow‐up remain the most effective tools for reducing CRC burden at the population level. At the same time, blood‐based screening, including FDA‐approved cfDNA testing in the United States, has expanded implementation options in selected settings, ctDNA has strengthened postoperative risk stratification, and biomarker‐directed therapy has continued to refine the management of metastatic disease. In selected biologic contexts, the implications are especially profound: organ‐preserving immunotherapy in localized dMMR disease has challenged conventional assumptions about mandatory surgery, while KRAS G12C‐, BRAF‐, and HER2‐directed strategies have shown that mechanistically informed combinations can convert previously resistant molecular subsets into actionable therapeutic groups.

However, the most important unmet needs remain substantial. A substantial biological and therapeutic gap remains between MSI‐high and microsatellite‐stable CRC, and most patients with MSS disease continue to derive limited benefit from current immunotherapy approaches. Likewise, although postoperative ctDNA has established strong prognostic value, its treatment‐guiding utility is stage‐, assay‐, timing‐, and intervention‐specific. Randomized Stage II data support selected reductions in chemotherapy use, whereas routine Stage III escalation or de‐escalation and treatment initiated solely at molecular recurrence have not yet been validated as standard strategies. Many promising innovations also risk deepening global inequity if they remain dependent on highly resourced centers with access to advanced endoscopy, molecular profiling, expert pathology, and intensive longitudinal surveillance.

Looking forward, the most consequential progress will likely come from linking mechanistic insight to pragmatic care pathways. Future research should prioritize three parallel goals: defining how tumor ecology and pathway crosstalk can be exploited to overcome resistance, particularly in MSS disease; translating real‐time biomarkers such as ctDNA and spatial or single‐cell profiling into clinically actionable algorithms; and ensuring that innovations in precision oncology are matched by innovations in access, implementation, and public‐health delivery. Progress in CRC will depend on integrating biology, clinical execution, and health‐system equity rather than advancing any one of them in isolation.

## Author Contributions

*Conceptualization*: Kairui Wan, Cheng Qian, and Limei Liu. *Literature investigation and evidence verification*: Kairui Wan, Ke Tang, Linglong Wang, Qin Tian, and Mingwei Zheng. *Writing – original draft*: Kairui Wan. *Writing – review and editing*: Kairui Wan, Cheng Qian, and Limei Liu. *Visualization*: Kairui Wan. *Supervision*: Cheng Qian and Limei Liu. The final manuscript has been reviewed and approved by all the authors.

## Ethics Statement

The authors have nothing to report.

## Conflicts of Interest

The authors declare no conflicts of interest.

## Data Availability

No new data were generated or analyzed in support of this review.
